# Immigration, mortality, and national life expectancy in the Nordic region, 1990–2019

**DOI:** 10.1016/j.ssmph.2022.101177

**Published:** 2022-07-31

**Authors:** Matthew Wallace, Michael J. Thomas, José Manuel Aburto, Anna Vera Jørring Pallesen, Laust Hvas Mortensen, Astri Syse, Sven Drefahl

**Affiliations:** a Stockholm University, Stockholm, Sweden; b Statistics Norway, Oslo, Norway; c University of Oxford, Oxford, England, UK; d University of Southern Denmark, Odense, Denmark; e University of Copenhagen, Copehagen, Denmark; f Statistics Denmark, Copehagen, Denmark; g Norwegian Institute of Public Health, Oslo, Norway

**Keywords:** MMA, Migrant Mortality Advantage, PLE, Period Life Expectancy, US, United States

## Abstract

• Immigrants have higher life expectancy at age 1 than the native-born in Denmark, Finland and Norway do from 1990 to 2019.
• Immigrants in Denmark, Finland and Norway increasingly enhance national life expectancy at age 1 over time.
• Immigrants in Sweden have lower life expectancy at age 1 than native-born in Sweden do in 1990, but similar levels by 2019.
• The effect of immigrants on national life expectancy at age 1 in Sweden transforms from negative to positive over time.
• The unique mortality of immigrants affects rankings of life expectancy at age 1 in the Nordic region in recent years.

## 1 Introduction

Period life expectancy is defined as the average number of additional years someone of a given age would live if current age-specific mortality rates were to stay the same for the remainder of their life. It is one of the world's most widely used population health metrics to summarize, compare and rank the mortality situation of countries, forming the basis for various public health, life insurance and retirement policies (Luy et al., 2020). This is because a country's life expectancy reflects, among other things, its existing socio-economic conditions and the quality of its public health and healthcare infrastructure (Ho & Hendi, 2018). Although immigration and emigration events are routinely factored into estimates of life expectancy, the potential impact of the unique – and typically low mortality – of international migrants (Aldridge et al., 2018; Shor & Roelfs, 2021) on national life expectancy has received little attention. A handful of studies have so far been limited to Australia (Page et al., 2007) and the United States (US) (Hendi & Ho, 2021; Mehta et al., 2016; Preston & Elo, 2014). In the context of rising shares of migrants in many countries (United Nations, 2019), alongside their “ageing in place” (Ciobanu et al., 2017), the extent to which the mortality of international migrants affects national population health demands attention.

Here, we aim to understand whether and how the mortality of international migrants affect the estimation and comparison of national life expectancy in four Nordic countries. Denmark, Finland, Norway and Sweden are all high-income countries in the latest stage of health transition. They have a long tradition of collaboration with shared features of social policy and universal welfare (Knudsen et al., 2019). Despite this, health inequality gaps remain large in the region relative to countries with less developed welfare systems (Mackenbach et al., 2016). International migration has been the major driving force behind population growth in the Nordic region in the past few decades. All four countries have experienced large increases in their absolute and relative numbers of migrants, alongside a transformation in migrant inflows from principally intra-Nordic flows to flows from all over the world (Karlsdottir et al., 2018). In recent years, life expectancy gains have slowed within the region; national life expectancies have also converged, although men and women in Denmark and men in Finland continue to lag some way behind Norway and Sweden (Knudsen et al., 2019).

To achieve our aim, we pose the following research questions:

**Table:** 

**RQ1:** What direction and size of effect, if any, does the mortality of international migrants have on national life expectancy in Denmark, Finland, Norway, and Sweden? **RQ2:** How does the effect of the mortality of international migrants on national life expectancy change over time? **RQ3:** Is there a specific gender effect to the influence of international migrants on national life expectancy? **RQ4:** Does the effect of the mortality of international migrants on national life expectancy affect comparisons and rankings of mortality within the Nordic region?

## 2 Background

### 2.1 Migrant mortality advantage

The “migrant mortality advantage” (MMA) refers to the lower mortality of migrants relative to the native-born population of the host country that they reside in (Guillot et al., 2018). Over the past several decades, this phenomenon has been exhaustively documented, particularly in high-income countries (Aldridge et al., 2018; Shor & Roelfs, 2021). MMA research displays certain commonalities. Those moving between high-income countries rarely exhibit an MMA, while those moving from low and middle-income to high-income countries consistently exhibit substantial MMAs over the native-born population (Shor & Roelfs, 2021). The MMA also displays a distinctive age pattern of mortality. Relative to the native-born population, migrants experience elevated mortality during childhood, a substantial “U-shape” of mortality advantage at young adult ages, followed by a gradual mortality convergence into older ages (Guillot et al., 2018; Wallace & Wilson, 2022). Typically, the MMA attenuates with length of residence in the host country (Hajat et al., 2010; Syse et al., 2018; Vandenheede et al., 2015; Wallace et al., 2019).

Previously, the MMA has been shown to enhance life expectancy at birth by 0.30 and 0.50 years (in men) and by 0.20 and 0.40 years (in women) in Australia from 1981 to 2000 (Page et al., 2007). It has also been shown to enhance life expectancy at age one by 0.32–0.94 years (in men) and by 0.26–0.83 years (in women) from 1990 to 2017 in the US (Hendi & Ho, 2021) and by 0.20 years (for both men and women) for life expectancy at age 65 (Mehta et al., 2016).

### 2.2 Explanatory mechanisms

Explanations of the MMA include the **healthy migrant effect**. It suggests that the low mortality of migrants is generated by strong selection forces that act directly on good health and indirectly on characteristics linked to good health (e.g., education level) (Wallace & Wilson, 2019). The **cultural factors hypothesis** posits that certain migrants come from countries where normative behaviours *promote* health, generating an MMA in those host countries where normative behaviours *erode* health (Guillot et al., 2018). The **salmon bias hypothesis** proposes that migrants in poor health are more likely to return to their origin country than migrants in good health are. Consequently, only healthier migrants who stay in the host country are included in calculations of mortality and the resulting estimates are not reflective of all those who moved (Turra & Elo, 2008). The **data artefact hypothesis** states that the MMA is merely a product of several data issues inherent to international migrant populations – one that is highly mobile and difficult to capture in data sources (Guillot et al., 2018). These include under-coverage of deaths (due to a higher possibility of death abroad) and over-coverage of the population (as migrants may remain registered in the host country but are no longer living there) (Monti et al, 2020).

### 2.3 History of migration in the Nordic region

Immigration in the Nordic region prior to the 1980s was characterised by intra-Nordic flows, predominantly to Sweden. This began with the arrival of refugees from Denmark, Finland and Norway (as well as other affected European countries) during World War II (Karlsdottir et al., 2018). Following the war, and aided by the *1954 Common Nordic Labour Market* agreement – which permitted the free mobility of labour within the Nordic region – large numbers of low educated, blue-collar workers (mostly from Finland) began moving to Sweden (Pedersen et al., 2008). This represented the dominant migrant flow in the region up until the end of the 1970s, accounting for two thirds of all moves (Korkiasaari & Söderling, 2003). From the 1980s, intra-Nordic flows diminished and gradually balanced out, with Norway receiving a greater relative shares of Nordic migrants – albeit of a much lower absolute number of moves (Pedersen et al., 2008).

Nevertheless, there was *some* migration from outside the Nordic region before the 1970s, with targeted labour migration from countries including India, Greece, Morocco, Turkey and Yugoslavia to Denmark, Norway and Sweden (Bevelander et al., 2013). These migrant flows ended with the enforcement of labour migration stops in all three countries in the 1970s (Bevelander et al., 2013). Yet, this merely led to a change in the composition of new migrant flows, with subsequent inflows comprising family members of existing labour migrants and refugees from countries including Chile, Vietnam (1970s), Iran, Iraq, Ethiopia (1980s), Yugoslavia, Somalia (1990s), Iraq and Afghanistan (2000s) and Syria (2010s) (Karlsdottir et al., 2018). Finland's experience differs from that of Denmark, Norway and Sweden in that immigration was heavily restricted until the 1990s, at which point half of all migrants were foreign-born children of Finnish emigrants (Ansala et al., 2020). From 1990 onward, flows specific to Finland – from Russia and the Baltics – began to rise, alongside refugees from Yugoslavia and Somalia (Korkiasaari & Söderling, 2003). Since 2005, when the European Union (EU) accepted ten new member states, all four of the countries have witnessed an increase in flows of migrants from the EU (Pedersen et al., 2008).

### 2.4 Mortality among migrants in the Nordic region

Studies have observed low mortality among the migrant populations (as a whole) of Denmark (Norredam et al., 2012), Finland (Lehti et al., 2017) and Norway (Honkaniemi et al., 2017; Syse et al., 2016, 2018): high mortality has been observed among the migrant population (as a whole) of Sweden (Honkaniemi et al., 2017; Wallace & Wilson, 2022). Similar variation in mortality by origins has been documented across the countries. Relative to natives, high mortality is found among intra-Nordic migrants; low mortality is found among migrants from Western & Southern Europe and non-Western countries (except for Sub-Saharan Africa) (Honkaniemi et al., 2017; Lehti et al., 2017; Norredam et al., 2012; Syse et al., 2018; Wallace & Wilson, 2022). Nordic migrants have high mortality from cancers, circulatory diseases and external causes-of-death; the opposite is true for Western & Southern European and non-Western migrants (Honkaniemi et al., 2017; Lehti et al., 2017; Norredam et al., 2012; Wallace & Wilson, 2022). Migrant mortality is shown to increase with duration of residence and is elevated among migrants arriving as children (Juárez et al., 2018; Syse et al., 2018). Labour migrants experience the largest mortality advantages in the Nordic region. Nevertheless, refugee migrants still enjoy substantial mortality advantages over their respective native-born populations (Norredam et al., 2012; Syse et al., 2018).

### 2.5 Expectations

Based on our review, we expect migrants to enhance life expectancy in Denmark, Finland and Norway, but not in Sweden. Concerning trends over time, we expect the impact of migrants to increase over time as the relative share – and influence – of migrants on estimates of national life expectancy increases. However, any change over time would also be influenced by factors such as the changing origin composition of migrants and their average length of stay a given country. If migrants *are* to influence comparisons of national life expectancy in the Nordic region, they have the greatest potential to do so in recent years where national life expectancy levels have converged between countries.

## 3 Materials and methods

### 3.1 Register data and lifetables

The Nordic region is home to national administrative registries with comparable data structure and validity that facilitate reliable comparisons across countries (Maret-Ouda et al., 2017). We use the death and total population registers from each country to derive the death and population counts by year (1990–2019), age (in single years from 0 to 1 to the open-ended interval 95+), sex (male and female), and nativity status (foreign-born [i.e., international migrant] and native-born).

For deaths, in a calendar year we calculate the exact age-at-death for those individuals who die (i.e., $\frac{date\;of\;birth-date\;of\;death}{365.25}$), create a death indicator for each individual (i.e., 1 = died, 0 = alive), and then aggregate the number of deaths according to individuals’ age, sex and nativity status.

For the population counts, we construct a dichotomous variable indicating residence or not in each country at the end of a calendar year (i.e., 1 = resident, 0 = not resident) according to age, sex and nativity status. Whether or not someone is resident is determined in a comparable way across the four countries using trace evidence from multiple register data sources (Maret-Ouda et al., 2017). From these population counts, we derive midyear population estimates (i.e., $\frac{\sum people\;aged\;x\;in\;year\;t+\sum people\;aged\;x\;in\;year\;t+1}{2}$), which provide an indication as to how many people are living in a country during a calendar year, accounting for births, deaths and migration events.

Next, we derive age-specific death rates by sex and nativity status using the death counts and midyear estimates (i.e., $\frac{deaths\;at\;age\;x\;in\;year\;t}{midyear\;population\;at\;age\;x\;in\;year\;t}$). Lastly, the age-specific death rates and midyear estimates are fed into the *R* package *Demography* (see Hyndman et al. (2019) for relevant documentation) to generate the period lifetables. The calculations that form the basis of the lifetable function in R package *Demography* can be found in (Chiang, 1984; Keyfitz & Caswell, 2005; Preston et al., 2001). Supplementary file 1 provides an example lifetable from Sweden for those unfamiliar with this particular method.

From the lifetables, we take the life expectancy at age one (PLE*1*) of the total, native-born and migrant populations of each country by sex. Then, we calculate the difference between (a) the PLE*1* of migrants and native-born and (b) the PLE*1* of the total population and native-born. We operationalise the latter to quantify the effect of migrants on national life expectancy. PLE*1* is a more accurate measure than life expectancy at birth (PLE*0*) when studying migrants because so few migrants arrive within the first 28 days of life – when the risk of infant death is highest – that observing any deaths before age one would be incredibly unlikely (Hendi & Ho, 2021). Supplementary file 2 shows that PLE*0* results in an inflated difference between the longevity of migrants and native-born populations. However, it does not lead to an inflated difference between the life expectancy of the total and native-born populations (i.e., the main metric of interest in this study), precisely *because* there are so few deaths and person-year contributions of migrants before age 1. Thus, we refer readers who are interested in the more established population health measure PLE*0* – who want to compare the findings to other studies and national figures – to supplementary file 2.

### 3.2 Data quality

The quality of the Nordic national registers is very high (Maret-Ouda et al., 2017). Nevertheless, research *has* shown that the registers (and particularly migrants) are susceptible to population over-coverage. Monti et al. (2020) showed that the share of migrants registered in Sweden but no longer living there had slowly risen from 2% to 5% between 1990 and 2012. When correcting the mortality rates of migrants for over-coverage, they reported a sizeable effect at young adult ages; mortality was 1.2-1.5x higher in the corrected versus uncorrected rate (Monti et al., 2020). Concerning the under-coverage of deaths, the deaths of residents abroad have been included as part of the registers of Finland, Norway and Sweden since the past decade; Denmark does not record the deaths of residents abroad (except for deaths in Greenland and the Faroe Islands) (Laugesen et al., 2021). Thus, if migrants are more likely to spend time abroad than native-born, it may be that some migrant deaths are missed in earlier years. Studies relating to this – or its effect on migrant mortality – are lacking (Guillot et al., 2018).

Supplementary file 3 compares our estimates for the total resident population with the Human Mortality Database (HMD), a collection of high quality mortality data (Barbieri et al., 2015). We observe high consistency; our estimates are regularly within ± 0.05 years of the HMD for a given country, sex, and year. Between 1990 and 2009, the estimates for Finland are further from the HMD (+0.10–0.25) than anticipated. Consequently, when we compare the impact of the mortality of international migrants on national life expectancy rankings in the Nordic region (as per the analysis in Table 2), we only produce rankings from 2010 to 2019, when the difference between the Finnish estimates and the HMD is comparable to Denmark, Norway and Sweden.

## 4 Results

Table 1 shows characteristics relating to the composition of the populations of the countries in 1990 and 2019. The sizeable increase in the absolute numbers of migrants in all four countries, combined with only a small increase among native-born people, shows how international migration has been driving population growth in the Nordic region. The relative shares of migrants have risen over time in all four countries. Nevertheless, Denmark and Finland remain below the high-income country average of 14.5% (United Nations, 2019). Larger relative shares of migrants in Norway and Sweden suggest a greater potential for migrants to affect national life expectancy levels in these two countries. Sweden's migrant population has (by far) the highest median age of the migrant populations in 1990, which is perhaps indicative of its longer migration history. However, it has also aged the least over the past three decades (unlike the migrant populations of Denmark, Finland and Norway). The narrow – and narrowing – interquartile range of all four migrant populations reflects the continued large-scale arrival of new migrants at peak migration ages. Danish and Finnish men and women have climbed the global life expectancy rankings from 1990 to 2019, Norwegian and Swedish men and women have fallen down the rankings. This speaks to the convergence of national life expectancy in the Nordic region in the past few decades.

**Table 1:** Population, migration, and mortality characteristics of the four countries.

	Denmark		Finland		Norway		Sweden	
	1990	2019	1990	2019	1990	2019	1990	2019
Total resident population								
n	5,141,155	5,771,876	4,996,222	5,532,156	4,247,285	5,378,857	8,567,384	10,036,379
Native-born population								
n	4,905,966	5,048,998	4,932,967	5,149,040	4,054,698	4,511,092	7,778,617	8,031,169
%	95.4	87.5	98.7	93.1	95.5	83.9	90.8	80.0
Median age, IQR	39 (37)	44 (44)	36 (34)	43 (41)	35 (38)	40 (41)	38 (38)	42 (42)
Foreign-born population								
n	235,189	722,878	63,255	383,116	192,587	867,765	788,767	2,005,210
%	4.6	12.5	1.3	6.9	4.5	16.1	9.2	20.0
Median age, IQR	32 (26)	41 (23)	26 (30)	36 (21)	32 (22)	37 (21)	40 (26)	41 (27)
United Nations world life expectancy ranking								
Men	32nd	29th	38th	24th	15th	16th	5th	9th
Women	35th	29th	17th	11th	12th	17th	6th	15th

**Table 2:** Life expectancy at age one “league table” for the total and native-born populations of the Nordic region, 2010-2019.

Year	Men							Women					
		Total population			Native-born				Total population			Native-born	
	Rank	Country	PLE*1*		Country	PLE*1*		Rank	Country	PLE*1*		Country	PLE*1*

**2010**	**1st**	Sweden	78.69	=	Sweden	78.76		**1st**	Sweden	82.67	=	Sweden	82.63
	**2nd**	Norway	78.09	=	Norway	78.03		**2nd**	Norway	82.35	=	Norway	82.26
	**3rd**	Denmark	76.38	=	Denmark	76.29		**3rd**	Finland	82.25	=	Finland	82.20
	**4th**	Finland	75.79	=	Finland	75.71		**4th**	Denmark	80.57	=	Denmark	80.53

**2011**	**1st**	Sweden	78.94	=	Sweden	78.98		**1st**	Sweden	82.81	=	Sweden	82.82
	**2nd**	Norway	78.22	=	Norway	78.09		**2nd**	Finland	82.60	=	Finland	82.55
	**3rd**	Denmark	76.96	=	Denmark	76.88		**3rd**	Norway	82.59	=	Norway	82.52
	**4th**	Finland	76.25	=	Finland	76.17		**4th**	Denmark	81.09	=	Denmark	81.01

**2012**	**1st**	Sweden	79.07	=	Sweden	79.13		**1st**	Sweden	82.71	=	Sweden	82.67
	**2nd**	Norway	78.63	=	Norway	78.53		**2nd**	Norway	82.58	=	Norway	82.49
	**3rd**	Denmark	77.31	=	Denmark	77.27		**3rd**	Finland	82.44	=	Finland	82.41
	**4th**	Finland	76.55	=	Finland	76.45		**4th**	Denmark	81.29	=	Denmark	81.22

**2013**	**1st**	Sweden	79.28	=	Sweden	79.36		**1st**	Sweden	82.91	=	Sweden	82.90
	**2nd**	Norway	78.82	=	Norway	78.68		**2nd**	Norway	82.83	↓	Finland	82.74
	**3rd**	Denmark	77.49	=	Denmark	77.41		**3rd**	Finland	82.78	↑	Norway	82.73
	**4th**	Finland	76.89	=	Finland	76.77		**4th**	Denmark	81.58	=	Denmark	81.55

**2014**	**1st**	Sweden	79.51	=	Sweden	79.53		**1st**	Norway	83.28	↓	Sweden	83.17
	**2nd**	Norway	79.23	=	Norway	79.10		**2nd**	Sweden	83.19	↑	Norway	83.13
	**3rd**	Denmark	77.92	=	Denmark	77.84		**3rd**	Finland	82.85	=	Finland	82.82
	**4th**	Finland	77.18	=	Finland	77.08		**4th**	Denmark	81.96	=	Denmark	81.93

**2015**	**1st**	Norway	79.57	↓	Sweden	79.53		**1st**	Norway	83.32	=	Norway	83.21
	**2nd**	Sweden	79.51	↑	Norway	79.37		**2nd**	Sweden	83.19	=	Sweden	83.17
	**3rd**	Denmark	78.07	=	Denmark	77.98		**3rd**	Finland	83.15	=	Finland	83.14
	**4th**	Finland	77.59	=	Finland	77.48		**4th**	Denmark	81.98	=	Denmark	81.95

**2016**	**1st**	Norway	79.79	↓	Sweden	79.73		**1st**	Norway	83.33	↓	Sweden	83.26
	**2nd**	Sweden	79.74	↑	Norway	79.63		**2nd**	Sweden	83.27	↑	Norway	83.15
	**3rd**	Denmark	78.22	=	Denmark	78.16		**3rd**	Finland	83.10	=	Finland	83.05
	**4th**	Finland	77.45	=	Finland	77.28		**4th**	Denmark	82.00	=	Denmark	81.98

**2017**	**1st**	Norway	80.09	=	Norway	79.97		**1st**	Norway	83.47	=	Norway	83.33
	**2nd**	Sweden	79.88	=	Sweden	79.88		**2nd**	Sweden	83.29	=	Sweden	83.27
	**3rd**	Denmark	78.41	=	Denmark	78.38		**3rd**	Finland	83.24	=	Finland	83.17
	**4th**	Finland	77.76	=	Finland	77.61		**4th**	Denmark	82.38	=	Denmark	82.31

**2018**	**1st**	Norway	80.19	=	Norway	80.07		**1st**	Norway	83.68	=	Norway	83.52
	**2nd**	Sweden	79.95	=	Sweden	79.94		**2nd**	Sweden	83.39	=	Sweden	83.35
	**3rd**	Denmark	78.33	=	Denmark	78.25		**3rd**	Finland	83.33	=	Finland	83.30
	**4th**	Finland	77.92	=	Finland	77.80		**4th**	Denmark	82.21	=	Denmark	82.15

**2019**	**1st**	Sweden	80.53	=	Sweden	80.48		**1st**	Sweden	83.90	=	Sweden	83.83
	**2nd**	Norway	80.39	=	Norway	80.25		**2nd**	Norway	83.82	=	Norway	83.66
	**3rd**	Denmark	78.70	=	Denmark	78.63		**3rd**	Finland	83.58	=	Finland	83.53
	**4th**	Finland	78.28	=	Finland	78.14		**4th**	Denmark	82.61	=	Denmark	82.56

Notes: Years with rank changes due to the PLE*1* contributions of international migrants are shaded in blue.

Source: authors’ calculations based upon respective register data for each country.

Supplementary file 4 displays the origin composition of migrants in 2019, including by length of stay. In all four of the countries in 2019, approximately half of all migrants have Western (and predominantly European) origins and half have non-Western (and predominantly Asian, which in this categorisation also includes Middle Eastern) origins. In Finland and Sweden, there is a clear gradient of *increasing* shares of non-Western migrants with *decreasing* length of stay. In Denmark and Norway, a similar trend can be seen for European Union (EU) and European Economic Area (EEA) migrants.

**Fig. 1:**
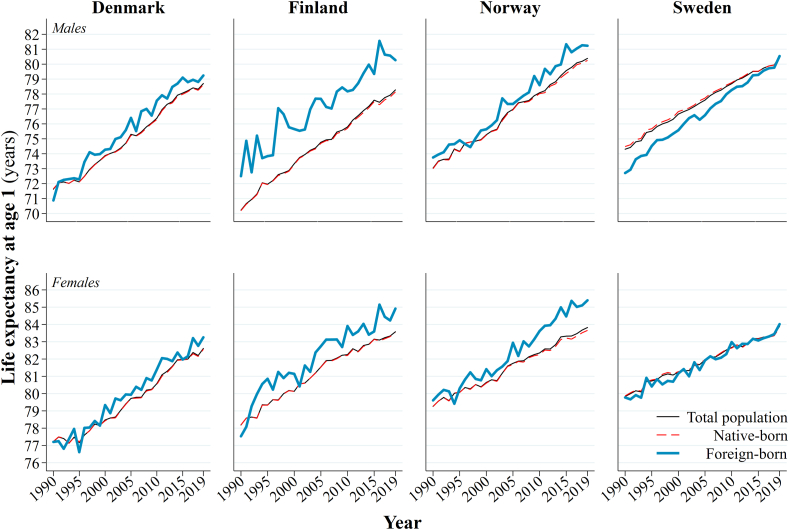
PLE*1* among men and women in Denmark, Finland, Norway, and Sweden, 1990–2019, *within* country comparisons of total, native-born, and international migrant populations.

Source: authors' calculations based upon the population registers of Denmark, Finland, Norway, and Sweden.

Fig. 1 presents long run PLE*1* trends for the total, native-born and migrant populations of Denmark, Finland, Norway, and Sweden from 1990 to 2019. The exact figures can be found in supplementary file 2. PLE*1* is almost universally higher among migrant men and women in Denmark, Finland and Norway compared to their respective native-born populations over time. The PLE*1* of native-born men and women is indistinguishable from the PLE*1* of the total population in these countries in 1990. However, a visible gap emerges by 2019 between the higher PLE*1* of all men and women and the lower PLE*1* of native-born men and women. The PLE*1* of migrant men and women in Sweden is lower than the PLE*1* of native-born men and women in 1990. Consequently, at the start of the period, the PLE*1* of native-born is visibly higher than the PLE*1* of the total population. Over time, however, the PLE*1* of migrant men and women accelerates faster than it does among the native-born population and the PLE*1* of migrants catches the PLE*1* of the native-born population by 2019.

**Fig. 2:**
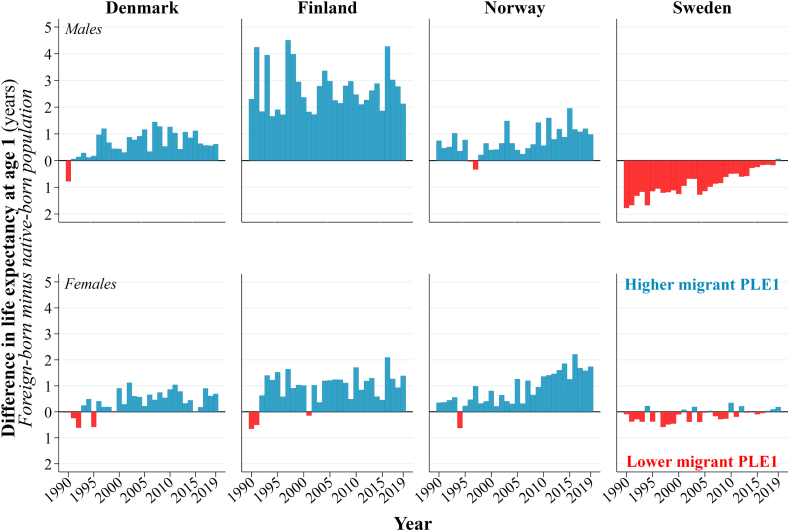
Differences in the PLE*1* of the migrant and native-born populations of Denmark, Finland, Norway, and Sweden, 1990–2019. Notes: PLE*1* (period life expectancy at age one). Source: authors' calculations based upon the population registers of Denmark, Norway, and Sweden.

Fig. 2 displays the difference in PLE*1* between migrant and native-born populations. The gap is largest among men in Finland, where the PLE*1* of migrant men is at least 2-years higher than the PLE*1* of native-born men. Among women in Finland and men and women in Denmark and Norway, the PLE*1* of migrants is half a year to 1-year higher than the native-born population. There is no clear trend in Denmark or Finland (i.e., the gap in PLE*1* is not gradually increasing or decreasing overtime). In Norway, the gap in PLE*1* is increasing gradually, especially among women. For men in Sweden, there is also a clear trend; PLE*1* is initially 1.5-years lower among migrants than it is among the native-born population. This gap converges then fully over time. For women in Sweden, PLE*1* is often lower among migrants compared to native-born women by approximately a third to a half of a year until 2015, after which it is similar to native-born women.

**Fig. 3:**
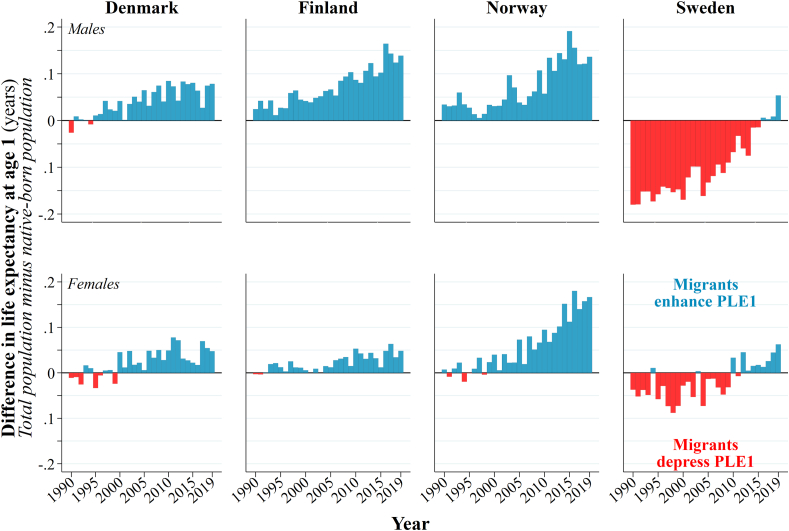
The effect of international migrants on PLE*1* in Denmark, Finland, Norway, and Sweden, 1990–2019. Notes: PLE*1* (period life expectancy at age one). Source: authors' calculations based upon the population registers of Denmark, Norway, and Sweden.

Fig. 3 shows the contributions of international migrants to national life expectancy. For men in Finland and Denmark, and men and women in Norway, migrants are increasingly enhancing national life expectancy over time. The size of the effect is largest among migrants in Norway in recent years, with peak impacts of +0.19 years among men (2015) and +0.18 years among women (2016), followed by men in Finland (+0.16 years in 2015). For men in Denmark, the size of the effect is smaller at +0.09 years (2013). While the size of these effects are modest, the increasing influence of migrants on life expectancy over time is clear. The impact of migrant women in Denmark and Finland is smaller – and the increase over time somewhat less evident. Nevertheless, both have a small positive impact on PLE*1* between 1990 and 2019. For Sweden, there is a major transformation in how international migrants influence national PLE*1*. Migrant men initially have a negative impact on PLE*1* in 1990 (−0.18 years) that gradually reduces and reverses to a modest positive impact in 2019 (+0.05 years). The same trend can be found for migrant women, with the peak effects instead coming in 1998 (−0.09 years) and 2019 (+0.06 years).

**Fig. 4:**
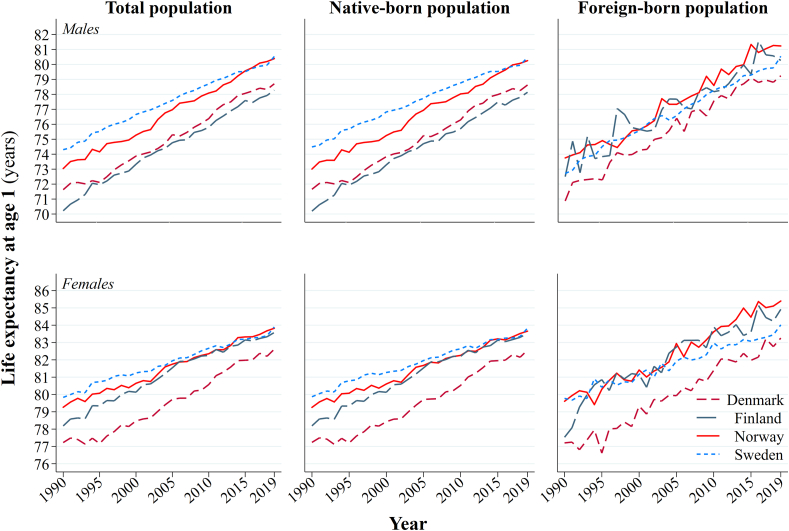
PLE*1* among men and women in Denmark, Finland, Norway, and Sweden, 1990–2019 *across* country comparisons of the total, native-born, and international migrant populations. Source: authors' calculations based upon the population registers of Denmark, Norway, and Sweden.

Fig. 4 rearranges the information from Fig. 1 by subpopulation to facilitate the direct comparison of the total, native-born and migrant populations of the Nordic region. National life expectancy has converged between the total populations of the countries between 1990 and 2019. Men in Norway, and women in Finland and Norway, have eliminated the gap to the traditional life expectancy leader of the region – Sweden. PLE*1* among men in Finland and Denmark, and women in Denmark, continues to lag behind the other countries. The trends for the native-born population are, expectedly, similar to those of the total population. Nevertheless, in recent years, as the PLE*1* of the native-born has converged, some small differences emerge relative to the total population that indicate some impact of migrants on comparisons of life expectancy within the region. Migrants in Denmark consistently have the lowest PLE*1* of the migrant populations, while migrants in Finland and Norway typically have some of the highest PLE*1*s in the Nordic region.

Table 2 shows the rankings of PLE*1* in the Nordic region from 2010 to 2019. In two of the ten years for men and three of the ten years for women, the rankings would have been different in the absence of migrants. The PLE*1* of men in Norway would have fallen behind men in Sweden in 2015 and 2016 (in both cases from 1st to 2nd place) without the enhancement of migrants to national PLE*1*. Moreover, the PLE*1* of women in Norway would have fallen behind women in Finland (in 2013, from 2nd to 3rd) and Sweden (in 2014 and 2016, from 1st to 2nd). In the past decade, migrants have helped Norway cement its role as a life expectancy leader in the Nordic region.

Supplementary file 5 shows the remaining life expectancy at age 25 (PLE*25*), age 50 (PLE*50*), and age 75 (PLE*75*) for the total, native-born and migrant populations of each country, along with the impact of migrants on remaining life expectancy at these ages. Migrant contributions at PLE*25* are almost identical to PLE*1*; halve in size by PLE*50* and then disappear by PLE*75*. Supplementary file 6 displays age-specific death rate ratios among migrants relative to native-born. The patterns in Denmark, Finland and Norway remain stable between 1990 and 2019 and are highly consistent with Guillot et al. (2018). Migrants have a relative excess mortality in childhood, a pronounced “U-shape” of mortality advantage at young adult ages, followed by a gradual mortality convergence into older ages. In Sweden, mortality among migrant men and women is systematically elevated over age in the 1990s. While the relative excesses in childhood and older age mortality remain in the 2010s, we document the clear emergence of a “U-shape” of mortality advantage over time among young adult migrants in Sweden between 1990 and 2019.

## 5 Discussion

In this study, our aim was to understand whether the estimation and comparison of national life expectancy in four countries of the Nordic region was being influenced by the unique mortality patterns of their international migrant populations. We set out to answer four specific research questions:

**RQ1** asked, “*What direction and size of effect, if any, does the mortality of international migrants have on national life expectancy in Denmark, Finland, Norway, and Sweden?*”. Migrants in Denmark, Finland and Norway modestly enhanced national life expectancy, while migrants in Sweden depressed national life expectancy in most years. **RQ2** asked, “*How does the effect of the mortality of international migrants on national life expectancy change over time?*”. Although the observed effect sizes were modest, migrants in Denmark, Finland and Norway increasingly enhanced national life expectancy over time. In Sweden, the effect of migrants reversed from a negative impact in 1990 to a minor positive one by 2019. **RQ3** asked, “*Is there a specific gender effect to the impact of international migrants on national life expectancy?*” The direction of the effect was consistent across the four countries for men and women. In Denmark and Finland there was a difference in the larger effect and clearer trend of migrant men on life expectancy. In Sweden there was a difference in the larger negative impact and clearer trend of men on life expectancy. **RQ4** asked, “*Does the effect of the mortality of international migrants on national life expectancy affect comparisons and rankings of mortality within the Nordic region?*” International migrants *are* beginning to influence comparisons and rankings of life expectancy in the Nordic region. The effect is most beneficial for Norway. Yet, it could reasonably be argued that this is facilitated by the close convergence of national life expectancies within the Nordic region in recent years, rather than a substantive effect of migrants. The reported findings largely fall in line with the expectations stated earlier on in the article.

### 5.1 Potential explanations

The findings are consistent with a **healthy migrant effect** and the transformation in migration flows in the countries from *negatively selected*, *higher mortality* intra-Nordic flows to *positively selected*, *lower mortality* EU and EEA and non-Western migrant flows between 1990 and 2019 (Honkaniemi et al., 2017; Lehti et al., 2017; Norredam et al., 2012; Syse et al., 2018; Wallace & Wilson, 2022). Intra-Nordic migrants, at least up until the 1980s, were negatively selected. In particular, the “mass migration” of Finns to Sweden from the 1950s–1970s comprised farmers and blue-collar workers with education far below the average of the Finnish population at the time (Pedersen et al., 2008). Although the direction of this selection reversed in the 1980s (with intra-Nordic migrants becoming more highly educated than their origin population), the number moving within the region had long since fallen (Pedersen et al., 2008). Given that Sweden was the main destination for intra-Nordic migrants up to the 1980s, this “*un*healthy migrant effect” could explain Sweden's departure from the similar patterns and trends of Denmark, Finland and Norway. The larger negative impact of migrant men (compared to women) on PLE*1* in Sweden in the early 1990s might reflect a stronger negative initial health selection among Finnish male migrants, increased hazards associated with the type of work they were doing in Sweden and/or higher smoking and alcohol prevalence among Finnish men (compared to women) (Östergren et al., 2019).

The gradual disappearance of this negative effect in Sweden – alongside the growing, positive impact of migrants in Denmark, Finland and Norway – might then be attributable to increasing inflows of recently arrived non-Western migrants in all four countries and EU/EEA migrants in Denmark and Norway (see supplementary file 4). Non-Western migrants should be strongly and positively selected due to the greater physical and cultural distance between their origin countries and the Nordic region, which is linked with higher moving costs and increased human capital (Chiswick et al., 2008; Shor & Roelfs, 2021). Unlike intra-Nordic migrants who can move without restrictions through the *Common Nordic Labour Market*, non-Western migrants are subject to stricter immigration controls (Pedersen et al., 2008). Although sizeable shares of non-Western migrants arrive in the Nordic countries under asylum – a reason for arrival not typically associated with strong selection effects (Chiswick et al., 2008) – non-Western refugees are shown to have substantial MMAs in the Nordic region (Norredam et al., 2012; Syse et al., 2018). EU/EEA flows, like intra-Nordic flows, also benefit from *freedom-of-movement* rights. Despite this, they might also be positively selected due to the reason for arrival, with many arriving to participate in tertiary education or highly skilled sectors of the labour market (Mooyaart & de Valk, 2020). Relative to native-born, the share of EU migrants with a tertiary education is higher in Denmark and Sweden and comparable in Norway (Eurostat, 2021). The more recent arrival of non-Western and EU/EEA migrants should mean that strong selection effects are still in force, resulting in pronounced MMAs at young adult ages (as supplementary file 6 shows) (Juárez et al., 2018; Syse et al., 2018).

The findings are also consistent with the **cultural factors** hypothesis. The transformation in migration flows in the Nordic region from 1990 to 2019 should translate into a gradual decrease in the relative shares of intra-Nordic migrants (*who have resided in the host country for a longer time and should be highly adapted*), alongside a gradual *increase* in the relative shares of non-Western migrants (*who have resided in the host country for a shorter time and should be less adapted*). This should shift the epidemiological profile of the migrant populations of the Nordic countries away from the risks factors (e.g., smoking, drinking and a high fat, high sugar diet) and diseases (e.g., chronic diseases) associated with high-income countries and toward those associated with low and middle-income countries. Crucially, however, non-Western migrants are theorised to undergo a “rapid-health-transition” when moving to high-income countries that instantly mitigates some of the risk factors (e.g. low healthcare quality and poor hygiene and sanitary conditions) and diseases (e.g., infectious diseases) that were previously important in the origin country (Spallek et al., 2011). Thus, migrants might arrive with a low mortality risk from the leading causes-of-death in the origin country *and* a low mortality risk for the leading causes-of-death in the host country (due to low exposure to the aforementioned risk factors associated with higher chronic disease risk) (Spallek et al., 2011). This shift could be crucial in the Nordic countries, where smoking, alcohol use (particularly among men in Denmark and Finland – which might help to explain the greater impact of migrant men compared to migrant women on PLE*1* in these countries) and metabolic risk factors are key risk factors and cancers and cardiovascular diseases are leading causes-of-death (Knudsen et al., 2019). Non-Western migrants in the Nordic region have very low cancer and cardiovascular mortality (Norredam et al., 2012; Honkaniemi et al., 2017; Wallace, 2021; Norredam et al., 2014), whereas Nordic migrants (notably Finns) have excess mortality from smoking-related, alcohol-related and cardiovascular diseases (Honkaniemi et al., 2017; Östergren et al., 2019; Wallace, 2021).

It seems unlikely that the growing impact of migrants on national life expectancy over time in the four countries is due to an increasing susceptibility to a **salmon bias effect** over time. This is because research from Denmark and Sweden documents an inverse association between emigration and health. Specifically, a *decreasing* risk of emigration is associated with *increasing* disease severity in Denmark (Norredam et al., 2014) and *increasing* comorbidity in Sweden (Dunlavy et al., 2022). There *is* partial evidence of an effect for specific origins. Migrants from Eastern Europe, the Middle East, Sub-Saharan Africa, and South Asia have a higher risk of emigration with low to moderate disease severity in Denmark (Norredam et al., 2014) and low to moderate comorbidity in Sweden (Dunlavy et al., 2022). However, migrants from the same origins with the highest comorbidity and/or disease severity are all more likely to stay in Denmark and Sweden respectively (Norredam et al., 2014; Dunlavy et al. 2022).

Similarly, it seems unlikely that an increasing susceptibility of the four migrant populations to the main **data artefacts** could fully account for the findings. It is true that levels of over-coverage *have* slowly risen among migrants (at least in Sweden) in the last several decades; it is also true that migrant mortality rates *are* downwardly biased by population over-coverage (Monti et al., 2020). Nevertheless, a recent study shows that correcting for this bias can only account for 19% of the MMA among women and 25% among men (in Sweden) (Wallace & Wilson, 2022). These figures are considerably lower among non-Western migrants, who have the largest MMAs, combined with the lowest levels of over-coverage of all origins (Monti et al., 2020; Wallace & Wilson, 2022). Taken together, this suggests that at least some of the impact of migrants on national life expectancy is genuine and that the origin composition of the migrant populations in the Nordic region is slowly transitioning to one that is less susceptible to over-coverage. Moreover, it seems unlikely that under-coverage of deaths could completely account for the findings. The deaths of residents abroad are captured in the registers in the past decade or so (Laugesen et al., 2021). Yet, there is no noticeable interruption to the observed trends and the effect of migrants on PLE*1* continues its gradual increase (or its emergence in the case of Sweden).

Migrant-centric theories aside, the results also reflect mortality developments among the native-born populations within and across the four countries. For example, the comparable impact of migrant men in Finland and Norway – despite lower relative share of migrants in Finland and the similar PLE*1* performance of migrant men in both countries – looks to be attributable to the inferior mortality performance of native-born men in Finland versus native-born men in Norway. Further, the smaller impact of migrant women on PLE*1* in Finland compared to migrant men in Finland reflects the superior mortality performance of native-born women (compared to native-born men) and *not* the inferior performance of migrant women (compared to migrant men). With respect to both of these observations, higher rates of cardiovascular disease, suicide, accident and alcohol deaths are responsible for the lower life expectancy of Finnish men compared to men in other Nordic countries and Finnish women (Knudsen et al., 2019). Finally, the main trends in Sweden owe as much to a *deceleration* in life expectancy gains among the native-born as they do to an *acceleration* in gains among migrants (that is sharper among men). Sweden is losing ground in life expectancy because mortality at higher ages has improved more slowly than in other countries, notably due to trends in cardiovascular disease mortality (Drefahl et al., 2014).

### 5.2 Research-in-context

That migrants enhance national life expectancy in Denmark, Finland, and Norway is consistent with evidence from Australia (Page et al., 2007) and the US (Hendi & Ho, 2021; Preston & Elo, 2014). However, the magnitude of the effects in the Nordic region is much smaller. Why? At least in Australia, the greater relative share of migrants as a share of the overall population compared to the countries of the Nordic region (United Nations, 2019) permits migrants a more influential role in the estimation of national life expectancy. However, the same is not true for the US (United Nations, 2019), where migrants have the largest observed effect on national life expectancy.

Next, it might be that the absolute difference in life expectancy between migrants and native-born is smaller in the Nordic region because *migrants* in the Nordic region do not live as long, on average, as those in Australia and the US. We might consider how differences in migration policy have affected the selectivity of migrant inflows and the size of the MMA. Specifically, Australia's points-based system (which is conditional on education level and skills) or the US green card system (in which all potential immigrants are subjected to an interview and medical examination) (Chiswick et al., 2008). We might additionally consider differences in the origin composition of migrant inflows and how they influence the selectivity, risk factors and disease prevalence of migrants. In the US, eight of every ten migrants had non-Western origins in 2019 (Budiman et al., 2020), compared to five in ten in the Nordic region (see supplementary file 4). Thus, a greater relative share of migrants in the US come from more culturally distant countries at an earlier stage of health transition, where the epidemiological profile is vastly different to the US. Half of non-Western migrants in the US also come from Latin America (Budiman et al., 2020); migrants from this set of countries have been shown to experience some of the most consistent and largest migrant mortality advantages of any origin-host combination (Ruiz et al., 2013).

Finally, it might also be that the absolute difference in life expectancy between migrants and native-born is smaller in the Nordic region because the *native-born* there do not live as long as the native-born populations of Australia and the US do. This is not the case for Australia, which has consistently rivalled Norway and Sweden in international male and female life expectancy rankings (Page et al., 2007). However, this *could* be the case for the US. Life expectancy in the US began to fall behind other rich countries in the 1980s and has continued to fall further behind ever since (Barbieri, 2019). Hendi and Ho (2021) showed that, between 2010 and 2017, while migrants continued to enjoy life expectancy gains, the US-born population showed consecutive declines, further amplifying the positive effect of migrants.

### 5.3 Conclusions

Strengths of the article include the use of high-quality register data of similar structure, validity and quality and (for the first time in the literature) the adoption of an international comparative perspective to study the influence of the unique mortality risks of migrants on a recognisable national population health measure across several decades. Weaknesses of the study include an analysis conducted at the arithmetic level, alongside a lack of correction for death under-coverage and population over-coverage (which suggests that the effects might be even smaller than the ones ' documented here). Future research could look beyond the arithmetic level to decompose the impact of migrants on life expectancy in the Nordic region by factors such as age, the country of origin and cause-of-death.

The unique mortality of international migrants is having a growing impact on the estimation of national life expectancy in the Nordic region that varies in size and direction across countries. While the effect sizes are modest, migrants *are* beginning to affect international comparisons and rankings of life expectancy. Crucially, we have demonstrated, in contrast with the existing evidence base, that migrants do not always enhance national life expectancy (i.e., in Sweden). The same cannot be said for Norway, Denmark and Finland, where continued improvements in national population health are at least partially attributable to immigrants. The stable trends point to the continuation of this growing effect of migrants on national life expectancy in Denmark, Finland and Norway, and the continued emergence of a positive impact in Sweden. Nonetheless, any future evolutions will also depend on changes in the size and composition of migrant flows, ongoing health transitions in major migrant sending countries, and continued mortality developments among the native-born populations of the Nordic region. National and international organisations that routinely publish life expectancy estimates should begin to consider how this increasing, dynamic and differential impact of migrants might start to affect the ability to compare life expectancy estimates over time within and across countries.

## Ethical approval

Stockholm Regional Committee for Research Ethics (dnr. 2017/1623-31/5).

## Funding

The 10.13039/501100006636Swedish Research Council for Health, Working Life and Welfare (*Forte*); grants: 2019-00603 ‘*Migrant mortality advantage lost? Emerging lifespan inequalities among migrants and their descendants in Sweden*’; 2016–07105 ‘*Migrant Trajectories*’; and 2016–07115 ‘*Ageing Well*’. The Norwegian data analysis was supported by a grant from the 10.13039/501100005416Norwegian Research Council (#256678). José Manuel Aburto was supported by the British Academy's Newton International Fellowship (NIFBA19/190679). Laust Hvas Mortensen is supported by the 10.13039/501100009708Novo Nordisk Foundation (NNF17OC0027594, NNF17OC0027812). Anna Vera Jørring Pallesen is supported by grants from the 10.13039/501100009708Novo Nordisk Foundation (NNF17OC0027812) and Centre for Health Aging, 10.13039/501100001734University of Copenhagen. The funding sources mentioned in this statement played no role in the study design; in the collection, analysis, and interpretation of the data; in the writing of the final report; or in the decision to submit the paper for publication. We confirm that we had full access to all of the data in this study and accept the responsibility to submit for publication.

## Data sharing

The individual-level register data for all of these countries, which was used to build national-level death and population data for input into lifetables is only available to named researchers following approval on various projects (including those provided above). Therefore, access to the data required to replicate these analyses is only available through participation in relevant projects.

## Transparency

The lead author (MW) affirms that the work described has not been published previously, that it is not under consideration for publication elsewhere, that its publication is approved by all authors and that, if accepted, it will not be published elsewhere in the same form, in English or in any other language, including electronically without the written consent of the copyright-holder.

## Author contributions

Matthew Wallace (MW) and Astri Syse (AS) conceived the study. MW was responsible for data access, preparation, and analysis in Sweden. Sven Drefahl (SD) was responsible for data access, preparation, and analysis in Finland. AS and Michael J Thomas (MJT) were responsible for data access and preparation in Norway; MW was responsible for data analysis in Norway. José Manuel Aburto (JMA), Anna Vera Jørring Pallesen (AVJP), and Laust Hvas Mortensen (LHM) were responsible for data access, preparation, and analysis in Denmark. MW drafted the paper; AS, SD, MJT, JMA, AVJP, and LHM offered comments and changes to subsequent drafts.

## Declaration of competing interest

None to declare.

## Data Availability

The authors do not have permission to share data.
