# Supplementary material for: Immigration, mortality, and national life expectancy in the Nordic region, 1990–2019
Source: SSM Popul Health. 2022 Jul 31;19:101177. doi: 10.1016/j.ssmph.2022.101177 (PMC9421394; doi:10.1016/j.ssmph.2022.101177)
Supplement: Multimedia component 1 [file mmc1.pdf]

Supplementary file 1

Table 1. Example lifetable for men in Sweden in 2019

x	mx	qx	lx	dx	Lx	Tx	ex
0	0.0022	0.0022	1.0000	0.0022	0.9979	81.3495	81.3495
1	0.0001	0.0001	0.9978	0.0001	0.9977	80.3516	80.5316
2	0.0000	0.0000	0.9976	0.0000	0.9976	79.3539	79.5434
3	0.0001	0.0001	0.9976	0.0001	0.9975	78.3563	78.5472
4	0.0001	0.0001	0.9975	0.0001	0.9974	77.3588	77.5521
5	0.0001	0.0001	0.9974	0.0001	0.9973	76.3613	76.5618
6	0.0000	0.0000	0.9973	0.0000	0.9972	75.3640	75.5714
7	0.0000	0.0000	0.9972	0.0000	0.9972	74.3668	74.5749
8	0.0001	0.0001	0.9972	0.0001	0.9971	73.3696	73.5773
9	0.0000	0.0000	0.9971	0.0000	0.9971	72.3724	72.5818
10	0.0000	0.0000	0.9971	0.0000	0.9971	71.3753	71.5852
11	0.0001	0.0001	0.9970	0.0001	0.9970	70.3783	70.5874
12	0.0001	0.0001	0.9970	0.0001	0.9969	69.3813	69.5918
13	0.0000	0.0000	0.9969	0.0000	0.9969	68.3843	68.5974
14	0.0001	0.0001	0.9968	0.0001	0.9968	67.3875	67.6007
15	0.0002	0.0002	0.9967	0.0002	0.9967	66.3907	66.6075
16	0.0002	0.0002	0.9966	0.0002	0.9965	65.3940	65.6186
17	0.0003	0.0003	0.9964	0.0003	0.9963	64.3975	64.6307
18	0.0004	0.0004	0.9961	0.0004	0.9959	63.4013	63.6485
19	0.0005	0.0005	0.9958	0.0005	0.9955	62.4053	62.6713
20	0.0008	0.0008	0.9953	0.0008	0.9949	61.4098	61.7022
21	0.0006	0.0006	0.9945	0.0006	0.9942	60.4150	60.7488
22	0.0004	0.0004	0.9939	0.0004	0.9937	59.4208	59.7866
23	0.0005	0.0005	0.9934	0.0005	0.9932	58.4271	58.8127
24	0.0008	0.0008	0.9929	0.0008	0.9925	57.4339	57.8432
25	0.0008	0.0008	0.9921	0.0008	0.9917	56.4414	56.8889
26	0.0007	0.0007	0.9913	0.0007	0.9910	55.4497	55.9348
27	0.0004	0.0004	0.9906	0.0004	0.9905	54.4587	54.9727
28	0.0007	0.0007	0.9903	0.0007	0.9899	53.4682	53.9945
29	0.0006	0.0006	0.9896	0.0006	0.9893	52.4783	53.0311
30	0.0006	0.0006	0.9890	0.0006	0.9886	51.4890	52.0642
31	0.0007	0.0007	0.9883	0.0007	0.9880	50.5004	51.0962
32	0.0009	0.0009	0.9877	0.0009	0.9872	49.5124	50.1314
33	0.0004	0.0004	0.9867	0.0004	0.9865	48.5252	49.1772
34	0.0007	0.0007	0.9863	0.0007	0.9860	47.5387	48.1972
35	0.0007	0.0007	0.9856	0.0007	0.9853	46.5527	47.2324
36	0.0007	0.0007	0.9849	0.0007	0.9846	45.5674	46.2638
37	0.0008	0.0008	0.9842	0.0008	0.9838	44.5828	45.2976
38	0.0008	0.0008	0.9834	0.0007	0.9830	43.5990	44.3345
39	0.0008	0.0008	0.9827	0.0008	0.9823	42.6160	43.3678
40	0.0010	0.0010	0.9819	0.0010	0.9814	41.6337	42.4016
41	0.0008	0.0008	0.9809	0.0008	0.9805	40.6523	41.4456
42	0.0011	0.0011	0.9801	0.0011	0.9795	39.6719	40.4794
43	0.0010	0.0010	0.9790	0.0010	0.9785	38.6924	39.5234
44	0.0011	0.0011	0.9780	0.0011	0.9775	37.7139	38.5619
45	0.0013	0.0013	0.9769	0.0013	0.9763	36.7364	37.6049
46	0.0015	0.0015	0.9756	0.0014	0.9749	35.7601	36.6541
47	0.0011	0.0011	0.9742	0.0010	0.9737	34.7852	35.7071
48	0.0015	0.0015	0.9731	0.0014	0.9724	33.8116	34.7449
49	0.0016	0.0016	0.9717	0.0015	0.9709	32.8392	33.7956
50	0.0019	0.0019	0.9702	0.0019	0.9692	31.8682	32.8487

51	0.0019	0.0019	0.9683	0.0018	0.9674	30.8990	31.9105
52	0.0023	0.0023	0.9665	0.0023	0.9653	29.9316	30.9699
53	0.0027	0.0027	0.9642	0.0026	0.9629	28.9663	30.0411
54	0.0029	0.0029	0.9616	0.0028	0.9602	28.0034	29.1223
55	0.0035	0.0035	0.9588	0.0034	0.9571	27.0432	28.2045
56	0.0040	0.0040	0.9555	0.0038	0.9536	26.0860	27.3017
57	0.0043	0.0043	0.9517	0.0041	0.9496	25.1324	26.4083
58	0.0042	0.0042	0.9476	0.0040	0.9456	24.1828	25.5211
59	0.0050	0.0050	0.9436	0.0047	0.9412	23.2373	24.6274
60	0.0059	0.0059	0.9388	0.0055	0.9360	22.2961	23.7492
61	0.0061	0.0061	0.9333	0.0057	0.9304	21.3600	22.8872
62	0.0077	0.0077	0.9276	0.0071	0.9241	20.4296	22.0239
63	0.0078	0.0078	0.9205	0.0072	0.9169	19.5055	21.1904
64	0.0084	0.0084	0.9133	0.0077	0.9095	18.5886	20.3526
65	0.0100	0.0099	0.9057	0.0090	0.9012	17.6791	19.5209
66	0.0114	0.0113	0.8967	0.0102	0.8916	16.7780	18.7117
67	0.0116	0.0116	0.8865	0.0103	0.8814	15.8864	17.9203
68	0.0136	0.0135	0.8763	0.0118	0.8703	15.0050	17.1240
69	0.0144	0.0143	0.8644	0.0124	0.8583	14.1347	16.3512
70	0.0159	0.0158	0.8521	0.0134	0.8453	13.2764	15.5816
71	0.0166	0.0165	0.8386	0.0138	0.8317	12.4311	14.8233
72	0.0206	0.0204	0.8248	0.0168	0.8164	11.5994	14.0635
73	0.0214	0.0212	0.8080	0.0171	0.7995	10.7830	13.3453
74	0.0239	0.0236	0.7909	0.0187	0.7816	9.9835	12.6229
75	0.0278	0.0275	0.7722	0.0212	0.7616	9.2020	11.9160
76	0.0301	0.0297	0.7510	0.0223	0.7399	8.4403	11.2383
77	0.0333	0.0327	0.7287	0.0238	0.7168	7.7004	10.5667
78	0.0362	0.0355	0.7049	0.0251	0.6924	6.9836	9.9072
79	0.0447	0.0437	0.6798	0.0297	0.6650	6.2912	9.2539
80	0.0487	0.0475	0.6501	0.0309	0.6347	5.6263	8.6539
81	0.0547	0.0533	0.6192	0.0330	0.6027	4.9916	8.0609
82	0.0631	0.0611	0.5863	0.0358	0.5683	4.3888	7.4862
83	0.0725	0.0700	0.5504	0.0385	0.5312	3.8205	6.9410
84	0.0818	0.0786	0.5119	0.0402	0.4918	3.2893	6.4258
85	0.0949	0.0906	0.4716	0.0427	0.4503	2.7976	5.9315
86	0.1102	0.1044	0.4289	0.0448	0.4065	2.3473	5.4726
87	0.1226	0.1155	0.3841	0.0444	0.3619	1.9408	5.0526
88	0.1413	0.1320	0.3397	0.0448	0.3173	1.5788	4.6472
89	0.1570	0.1456	0.2949	0.0429	0.2734	1.2615	4.2777
90	0.1872	0.1712	0.2520	0.0431	0.2304	0.9881	3.9212
91	0.1991	0.1811	0.2088	0.0378	0.1899	0.7577	3.6278
92	0.2363	0.2113	0.1710	0.0361	0.1530	0.5677	3.3196
93	0.2631	0.2325	0.1349	0.0314	0.1192	0.4148	3.0750
94	0.2925	0.2552	0.1035	0.0264	0.0903	0.2956	2.8552
95	0.3757	1.0000	0.0771	0.0771	0.2053	0.2053	2.6620

Source: authors' calculations based upon the Swedish register data "Ageing Well"

Column descriptions

**x**	Age (1-year interval; closed at ages 95+)
**mx**	Death rate at age x (i.e., number of deaths at age x in the calendar year divided by the mid-year population estimate at age x)
**qx**	Probability that an individual aged x will die before reaching age x+1
**lx**	Proportion of individuals (of 1) surviving to age x (who are assumed to be subject through their lives to the mortality rates experienced in year t)
**dx**	Proportion of individuals (of 1) dying between age x and age x+1
**Lx**	Number of years lived between age x and age x+1 (sums to e0)
**Tx**	Number of years lived from age x to the oldest age
**ex**	The average period life expectancy at age x i.e., the average number of further years individuals aged x will live based on the mortality rates experienced in year t

Supplementary file 2

Table 1. Robustness check of life expectancy at birth (PLE0) versus life expectancy at age 1 (PLE1), men, Denmark.

Year	Total population (a)		Native-born (b)		Foreign-born (c)		(a-b)		(b-c)	
	e0	e1	e0	e1	e0	e1	e0	e1	e0	e1
1990	71.99	71.63	72.01	71.65	71.88	70.88	-0.02	-0.03	0.13	0.78
1991	72.43	72.05	72.42	72.04	73.11	72.11	0.01	0.01	-0.69	-0.06
1992	72.54	72.11	72.54	72.11	73.25	72.25	0.01	0.00	-0.71	-0.14
1993	72.57	72.02	72.57	72.02	73.01	72.31	0.00	0.00	-0.44	-0.29
1994	72.75	72.23	72.76	72.24	72.52	72.36	-0.01	-0.01	0.25	-0.12
1995	72.70	72.12	72.68	72.11	73.28	72.28	0.01	0.01	-0.60	-0.18
1996	73.03	72.49	73.01	72.48	74.45	73.45	0.02	0.01	-1.44	-0.97
1997	73.56	72.95	73.52	72.91	74.79	74.11	0.04	0.04	-1.27	-1.20
1998	73.94	73.28	73.91	73.26	74.94	73.94	0.03	0.02	-1.02	-0.68
1999	74.19	73.55	74.17	73.53	74.98	73.98	0.02	0.02	-0.81	-0.45
2000	74.43	73.88	74.38	73.83	75.27	74.27	0.04	0.04	-0.89	-0.44
2001	74.66	74.02	74.66	74.02	75.32	74.32	0.00	0.00	-0.67	-0.31
2002	74.79	74.15	74.75	74.11	75.99	74.99	0.04	0.04	-1.24	-0.88
2003	75.00	74.38	74.95	74.33	76.11	75.11	0.05	0.05	-1.16	-0.78
2004	75.36	74.71	75.32	74.67	76.58	75.58	0.04	0.04	-1.26	-0.91
2005	75.91	75.30	75.85	75.24	77.40	76.40	0.07	0.06	-1.56	-1.16
2006	75.89	75.21	75.85	75.18	76.52	75.52	0.03	0.03	-0.66	-0.34
2007	76.12	75.46	76.05	75.40	77.84	76.84	0.06	0.06	-1.79	-1.45
2008	76.47	75.80	76.39	75.73	78.01	77.01	0.08	0.07	-1.62	-1.28
2009	76.83	76.07	76.79	76.03	77.56	76.56	0.04	0.04	-0.77	-0.53
2010	77.10	76.38	77.01	76.29	78.56	77.56	0.09	0.08	-1.54	-1.26
2011	77.69	76.96	77.62	76.88	78.55	77.92	0.07	0.07	-0.93	-1.04
2012	78.04	77.31	78.00	77.27	78.70	77.70	0.04	0.04	-0.70	-0.43
2013	78.24	77.49	78.16	77.41	79.48	78.48	0.08	0.08	-1.32	-1.07
2014	78.56	77.92	78.48	77.84	79.34	78.70	0.08	0.08	-0.86	-0.86
2015	78.75	78.07	78.67	77.98	80.10	79.10	0.08	0.08	-1.43	-1.12
2016	78.93	78.22	78.87	78.16	79.80	78.80	0.07	0.06	-0.93	-0.64
2017	79.08	78.41	79.05	78.38	79.95	78.95	0.03	0.03	-0.90	-0.57
2018	79.00	78.33	78.93	78.25	79.81	78.81	0.08	0.07	-0.88	-0.56
2019	79.43	78.70	79.36	78.63	79.87	79.24	0.08	0.08	-0.51	-0.62

Notes: PLE0 (life expectancy at birth); PLE1 (life expectancy at age 1); a-b = total population minus native-born; b-c = native-born minus foreign-born

Source: authors' calculations based upon the Danish register data

Table 2. Robustness check of life expectancy at birth (PLE0) versus life expectancy at age 1 (PLE1), women, Denmark.

Year	Total population (a)		Native-born (b)		Foreign-born (c)		(a-b)		(b-c)	
	e0	e1	e0	e1	e0	e1	e0	e1	e0	e1
1990	77.70	77.22	77.71	77.23	77.87	77.20	-0.01	-0.01	-0.16	0.03
1991	77.96	77.49	77.97	77.50	77.94	77.25	-0.01	-0.01	0.04	0.25
1992	77.93	77.40	77.96	77.43	77.81	76.81	-0.02	-0.03	0.15	0.62
1993	77.75	77.13	77.73	77.12	78.37	77.37	0.02	0.02	-0.63	-0.25
1994	78.08	77.48	78.07	77.47	78.96	77.96	0.01	0.01	-0.89	-0.49
1995	77.81	77.17	77.84	77.20	77.62	76.62	-0.03	-0.03	0.22	0.58
1996	78.22	77.60	78.22	77.61	79.02	78.02	0.00	-0.01	-0.79	-0.41
1997	78.46	77.85	78.46	77.85	79.04	78.04	0.01	0.01	-0.58	-0.19
1998	78.88	78.24	78.87	78.23	79.42	78.42	0.01	0.01	-0.54	-0.19
1999	78.88	78.15	78.90	78.17	79.15	78.15	-0.02	-0.02	-0.25	0.03
2000	79.11	78.47	79.06	78.43	80.34	79.34	0.05	0.05	-1.27	-0.91
2001	79.21	78.59	79.19	78.58	79.87	78.87	0.01	0.01	-0.67	-0.29
2002	79.32	78.64	79.27	78.59	80.72	79.72	0.05	0.05	-1.44	-1.12
2003	79.72	79.03	79.70	79.01	80.61	79.61	0.02	0.02	-0.91	-0.60
2004	80.07	79.40	80.05	79.38	80.95	79.95	0.02	0.02	-0.90	-0.57
2005	80.43	79.72	80.42	79.71	80.93	79.93	0.01	0.01	-0.51	-0.22
2006	80.51	79.79	80.46	79.74	81.39	80.39	0.05	0.05	-0.94	-0.66
2007	80.51	79.79	80.48	79.76	81.22	80.22	0.04	0.03	-0.74	-0.46
2008	80.91	80.20	80.86	80.15	81.90	80.90	0.05	0.05	-1.04	-0.75
2009	81.02	80.25	80.99	80.22	81.76	80.76	0.03	0.03	-0.78	-0.54
2010	81.32	80.57	81.27	80.53	82.01	81.39	0.05	0.05	-0.74	-0.87
2011	81.81	81.09	81.74	81.01	82.68	82.05	0.08	0.08	-0.94	-1.04
2012	82.02	81.29	81.95	81.22	83.01	82.01	0.07	0.07	-1.06	-0.79
2013	82.29	81.58	82.26	81.55	82.88	81.88	0.03	0.03	-0.62	-0.33
2014	82.67	81.96	82.64	81.93	83.38	82.38	0.03	0.03	-0.74	-0.45
2015	82.68	81.98	82.65	81.95	82.97	81.97	0.02	0.02	-0.32	-0.02
2016	82.77	82.00	82.75	81.98	83.16	82.16	0.02	0.02	-0.41	-0.18
2017	83.11	82.38	83.04	82.31	84.21	83.21	0.07	0.07	-1.17	-0.90
2018	82.94	82.21	82.89	82.15	83.76	82.76	0.06	0.05	-0.88	-0.61
2019	83.40	82.61	83.35	82.56	84.26	83.26	0.05	0.05	-0.90	-0.69

Notes: PLE0 (life expectancy at birth); PLE1 (life expectancy at age 1); a-b = total population minus native-born; b-c = native-born minus foreign-born

Source: authors' calculations based upon the Danish register data

Table 3. Robustness check of life expectancy at birth (PLE0) versus life expectancy at age 1 (PLE1), men, Finland.

Year	Total population (a)		Native-born (b)		Foreign-born (c)		(a-b)		(b-c)	
	e0	e1	e0	e1	e0	e1	e0	e1	e0	e1
1990	71.14	70.22	71.11	70.19	73.50	72.50	0.02	0.02	-2.39	-2.31
1991	71.59	70.66	71.55	70.62	75.87	74.87	0.04	0.04	-4.32	-4.25
1992	71.89	70.94	71.87	70.92	73.76	72.76	0.03	0.03	-1.89	-1.84
1993	72.25	71.30	72.21	71.26	76.21	75.21	0.04	0.04	-4.01	-3.95
1994	73.01	72.05	73.00	72.04	74.70	73.70	0.01	0.01	-1.71	-1.66
1995	72.91	71.96	72.89	71.93	74.84	73.84	0.03	0.03	-1.96	-1.91
1996	73.16	72.21	73.13	72.18	74.90	73.90	0.03	0.03	-1.77	-1.72
1997	73.57	72.60	73.51	72.54	78.05	77.05	0.06	0.06	-4.54	-4.51
1998	73.68	72.73	73.62	72.66	77.65	76.65	0.06	0.06	-4.03	-3.99
1999	73.84	72.87	73.79	72.83	76.78	75.78	0.04	0.04	-2.98	-2.95
2000	74.29	73.33	74.25	73.29	76.66	75.66	0.04	0.04	-2.41	-2.37
2001	74.69	73.75	74.65	73.71	76.54	75.54	0.04	0.04	-1.88	-1.83
2002	74.91	73.95	74.87	73.90	76.63	75.63	0.05	0.05	-1.76	-1.73
2003	75.20	74.23	75.15	74.18	76.86	76.97	0.05	0.05	-1.71	-2.79
2004	75.35	74.39	75.29	74.33	78.69	77.69	0.06	0.06	-3.40	-3.36
2005	75.72	74.77	75.65	74.70	78.68	77.68	0.07	0.07	-3.03	-2.98
2006	75.90	74.93	75.85	74.88	78.14	77.14	0.05	0.05	-2.29	-2.26
2007	75.95	74.96	75.86	74.88	78.03	77.03	0.08	0.08	-2.17	-2.15
2008	76.42	75.46	76.33	75.36	79.16	78.16	0.09	0.09	-2.83	-2.80
2009	76.55	75.58	76.45	75.47	79.44	78.44	0.10	0.10	-2.99	-2.97
2010	76.76	75.79	76.67	75.71	79.18	78.18	0.09	0.09	-2.51	-2.47
2011	77.21	76.25	77.13	76.17	79.28	78.28	0.08	0.08	-2.14	-2.10
2012	77.52	76.55	77.42	76.45	79.72	78.72	0.11	0.11	-2.30	-2.27
2013	77.86	76.89	77.74	76.77	80.39	79.39	0.12	0.12	-2.65	-2.62
2014	78.14	77.18	78.05	77.08	80.97	79.97	0.09	0.09	-2.92	-2.89
2015	78.56	77.59	78.46	77.48	79.74	79.35	0.10	0.10	-1.28	-1.86
2016	78.41	77.45	78.25	77.28	82.56	81.56	0.16	0.16	-4.31	-4.27
2017	78.72	77.76	78.58	77.61	81.64	80.64	0.14	0.14	-3.06	-3.02
2018	78.91	77.92	78.78	77.80	81.57	80.57	0.12	0.12	-2.79	-2.78
2019	79.23	78.28	79.10	78.14	81.27	80.27	0.14	0.14	-2.17	-2.13

Notes: PLE0 (life expectancy at birth); PLE1 (life expectancy at age 1); a-b = total population minus native-born; b-c = native-born minus foreign-born

Source: authors' calculations based upon the Finnish register data

Table 4. Robustness check of life expectancy at birth (PLE0) versus life expectancy at age 1 (PLE1), women, Finland.

Year	Total population (a)		Native-born (b)		Foreign-born (c)		(a-b)		(b-c)	
	e0	e1	e0	e1	e0	e1	e0	e1	e0	e1
1990	79.12	78.19	79.13	78.19	78.53	77.53	0.00	0.00	0.59	0.66
1991	79.52	78.58	79.53	78.59	79.08	78.08	0.00	0.00	0.45	0.51
1992	79.57	78.64	79.57	78.64	80.27	79.27	0.00	0.00	-0.71	-0.63
1993	79.54	78.59	79.52	78.57	80.97	79.97	0.02	0.02	-1.45	-1.40
1994	80.31	79.35	80.29	79.33	81.56	80.56	0.02	0.02	-1.27	-1.22
1995	80.27	79.34	80.25	79.33	81.86	80.86	0.01	0.01	-1.60	-1.53
1996	80.61	79.65	80.61	79.65	81.23	80.23	0.00	0.00	-0.62	-0.58
1997	80.60	79.63	80.58	79.61	82.25	81.25	0.03	0.03	-1.67	-1.64
1998	80.94	80.00	80.93	79.99	81.90	80.90	0.01	0.01	-0.97	-0.91
1999	81.14	80.18	81.13	80.17	82.20	81.20	0.01	0.01	-1.08	-1.03
2000	81.09	80.13	81.08	80.13	82.14	81.14	0.01	0.01	-1.06	-1.01
2001	81.54	80.57	81.54	80.56	81.42	80.42	0.00	0.00	0.12	0.15
2002	81.58	80.61	81.57	80.60	82.63	81.63	0.01	0.01	-1.05	-1.02
2003	81.87	80.90	81.87	80.90	82.26	81.26	0.00	0.00	-0.39	-0.36
2004	82.18	81.20	82.17	81.19	83.38	82.38	0.01	0.01	-1.21	-1.19
2005	82.51	81.54	82.50	81.53	82.83	82.74	0.01	0.01	-0.33	-1.21
2006	82.88	81.91	82.85	81.88	84.12	83.12	0.03	0.03	-1.27	-1.24
2007	82.88	81.92	82.85	81.89	84.13	83.13	0.03	0.03	-1.28	-1.24
2008	83.03	82.05	83.00	82.02	84.13	83.13	0.03	0.03	-1.14	-1.11
2009	83.18	82.22	83.16	82.21	83.69	82.69	0.02	0.01	-0.53	-0.49
2010	83.21	82.25	83.16	82.20	84.91	83.91	0.05	0.05	-1.75	-1.71
2011	83.56	82.60	83.51	82.55	84.40	83.40	0.04	0.04	-0.88	-0.84
2012	83.40	82.44	83.37	82.41	84.60	83.60	0.03	0.03	-1.22	-1.19
2013	83.76	82.78	83.71	82.74	85.04	84.04	0.04	0.04	-1.32	-1.30
2014	83.83	82.85	83.80	82.82	84.41	83.41	0.03	0.03	-0.61	-0.59
2015	84.13	83.15	84.12	83.14	84.59	83.59	0.01	0.01	-0.48	-0.46
2016	84.09	83.10	84.04	83.05	86.15	85.15	0.05	0.05	-2.11	-2.10
2017	84.22	83.24	84.15	83.17	85.44	84.44	0.06	0.06	-1.29	-1.27
2018	84.30	83.33	84.26	83.30	85.23	84.23	0.03	0.03	-0.97	-0.93
2019	84.56	83.58	84.51	83.53	85.92	84.92	0.05	0.05	-1.40	-1.39

Notes: PLE0 (life expectancy at birth); PLE1 (life expectancy at age 1); a-b = total population minus native-born; b-c = native-born minus foreign-born

Source: authors' calculations based upon the Finnish register data

Table 5. Robustness check of life expectancy at birth (PLE0) versus life expectancy at age 1 (PLE1), men, Norway.

Year	Total population (a)		Native-born (b)		Foreign-born (c)		(a-b)		(b-c)	
	e0	e1	e0	e1	e0	e1	e0	e1	e0	e1
1990	73.44	73.04	73.40	73.00	74.30	73.75	0.03	0.03	-0.90	-0.75
1991	74.01	73.51	73.98	73.48	74.95	73.95	0.03	0.03	-0.97	-0.47
1992	74.16	73.63	74.13	73.59	75.11	74.11	0.03	0.03	-0.98	-0.51
1993	74.23	73.65	74.17	73.59	75.16	74.62	0.06	0.06	-0.99	-1.03
1994	74.89	74.32	74.85	74.29	75.65	74.65	0.04	0.03	-0.80	-0.36
1995	74.79	74.16	74.76	74.13	75.39	74.91	0.03	0.03	-0.62	-0.78
1996	75.36	74.70	75.35	74.69	75.65	74.65	0.01	0.01	-0.30	0.04
1997	75.45	74.79	75.45	74.78	75.45	74.45	0.01	0.01	0.00	0.34
1998	75.50	74.85	75.49	74.83	75.61	75.05	0.01	0.01	-0.12	-0.21
1999	75.61	74.95	75.57	74.91	76.56	75.56	0.04	0.03	-0.99	-0.65
2000	75.95	75.28	75.92	75.24	76.64	75.64	0.03	0.03	-0.72	-0.40
2001	76.19	75.52	76.16	75.49	76.90	75.90	0.03	0.03	-0.74	-0.42
2002	76.39	75.64	76.35	75.60	77.25	76.25	0.05	0.04	-0.90	-0.65
2003	77.04	76.32	76.94	76.22	78.71	77.71	0.10	0.10	-1.77	-1.49
2004	77.49	76.76	77.42	76.69	78.33	77.33	0.07	0.07	-0.91	-0.65
2005	77.72	76.97	77.68	76.94	78.33	77.33	0.04	0.04	-0.66	-0.40
2006	78.12	77.41	78.08	77.37	78.62	77.62	0.04	0.03	-0.54	-0.25
2007	78.23	77.48	78.18	77.43	78.89	77.89	0.05	0.05	-0.72	-0.46
2008	78.31	77.57	78.24	77.50	79.11	78.11	0.06	0.06	-0.87	-0.61
2009	78.59	77.89	78.48	77.78	80.20	79.20	0.11	0.11	-1.72	-1.43
2010	78.85	78.09	78.79	78.03	79.60	78.60	0.06	0.06	-0.81	-0.57
2011	78.99	78.22	78.86	78.09	80.69	79.69	0.14	0.13	-1.83	-1.60
2012	79.41	78.63	79.30	78.53	80.32	79.32	0.11	0.11	-1.02	-0.80
2013	79.65	78.82	79.50	78.68	80.86	79.86	0.15	0.14	-1.36	-1.18
2014	80.02	79.23	79.89	79.10	80.98	79.98	0.13	0.13	-1.09	-0.88
2015	80.36	79.57	80.16	79.37	82.33	81.33	0.19	0.19	-2.17	-1.96
2016	80.60	79.79	80.44	79.63	81.80	80.80	0.16	0.16	-1.36	-1.17
2017	80.91	80.09	80.79	79.97	82.05	81.05	0.12	0.12	-1.26	-1.08
2018	81.00	80.19	80.88	80.07	82.27	81.27	0.12	0.12	-1.39	-1.20
2019	81.19	80.39	81.05	80.25	82.24	81.24	0.14	0.14	-1.18	-0.98

Notes: PLE0 (life expectancy at birth); PLE1 (life expectancy at age 1); a-b = total population minus native-born; b-c = native-born minus foreign-born

Source: authors' calculations based upon the Norwegian register data

Table 6. Robustness check of life expectancy at birth (PLE0) versus life expectancy at age 1 (PLE1), women, Norway.

Year	Total population (a)		Native-born (b)		Foreign-born (c)		(a-b)		(b-c)	
	e0	e1	e0	e1	e0	e1	e0	e1	e0	e1
1990	79.80	79.26	79.79	79.25	80.61	79.61	0.01	0.01	-0.81	-0.35
1991	80.10	79.56	80.11	79.57	80.94	79.94	-0.01	-0.01	-0.83	-0.37
1992	80.36	79.78	80.35	79.77	81.22	80.22	0.01	0.01	-0.87	-0.45
1993	80.24	79.59	80.22	79.57	81.13	80.13	0.02	0.02	-0.91	-0.56
1994	80.65	80.02	80.67	80.04	80.42	79.42	-0.02	-0.02	0.25	0.63
1995	80.82	80.07	80.82	80.07	80.68	80.30	0.00	0.00	0.14	-0.23
1996	81.06	80.35	81.05	80.34	81.81	80.81	0.01	0.01	-0.76	-0.47
1997	80.97	80.28	80.94	80.24	82.23	81.23	0.03	0.03	-1.29	-0.99
1998	81.25	80.52	81.25	80.53	81.85	80.85	0.00	0.00	-0.60	-0.32
1999	81.12	80.39	81.09	80.37	81.77	80.77	0.03	0.02	-0.68	-0.40
2000	81.38	80.65	81.34	80.61	81.99	81.41	0.04	0.04	-0.65	-0.81
2001	81.52	80.80	81.51	80.79	82.01	81.01	0.01	0.01	-0.50	-0.21
2002	81.46	80.75	81.42	80.71	82.35	81.35	0.04	0.04	-0.94	-0.64
2003	81.93	81.18	81.91	81.16	82.17	81.57	0.02	0.02	-0.26	-0.41
2004	82.34	81.59	82.31	81.57	82.88	81.88	0.02	0.02	-0.57	-0.31
2005	82.52	81.75	82.44	81.68	83.94	82.94	0.07	0.07	-1.50	-1.26
2006	82.66	81.88	82.64	81.86	82.88	82.18	0.02	0.02	-0.23	-0.32
2007	82.66	81.90	82.58	81.82	84.02	83.02	0.08	0.08	-1.44	-1.20
2008	82.95	82.13	82.90	82.08	83.73	82.73	0.05	0.05	-0.83	-0.65
2009	83.05	82.26	82.99	82.19	84.14	83.14	0.07	0.07	-1.16	-0.95
2010	83.15	82.35	83.05	82.26	84.28	83.62	0.09	0.09	-1.23	-1.36
2011	83.45	82.59	83.38	82.52	84.93	83.93	0.07	0.07	-1.55	-1.41
2012	83.40	82.58	83.31	82.49	84.96	83.96	0.09	0.09	-1.64	-1.46
2013	83.60	82.83	83.50	82.73	85.33	84.33	0.10	0.10	-1.83	-1.61
2014	84.10	83.28	83.94	83.13	85.99	84.99	0.15	0.15	-2.04	-1.86
2015	84.15	83.32	84.04	83.21	85.47	84.47	0.11	0.11	-1.43	-1.26
2016	84.17	83.33	83.98	83.15	86.37	85.37	0.18	0.18	-2.38	-2.21
2017	84.28	83.47	84.14	83.33	86.02	85.02	0.14	0.14	-1.88	-1.69
2018	84.49	83.68	84.34	83.52	86.11	85.11	0.16	0.16	-1.77	-1.59
2019	84.68	83.82	84.51	83.66	86.40	85.40	0.17	0.17	-1.88	-1.74

Notes: PLE0 (life expectancy at birth); PLE1 (life expectancy at age 1); a-b = total population minus native-born; b-c = native-born minus foreign-born

Source: authors' calculations based upon the Norwegian register data

Table 7. Robustness check of life expectancy at birth (PLE0) versus life expectancy at age 1 (PLE1), men, Sweden.

Year	Total population (a)		Native-born (b)		Foreign-born (c)		(a-b)		(b-c)	
	e0	e1	e0	e1	e0	e1	e0	e1	e0	e1
1990	74.79	74.30	74.97	74.48	73.27	72.71	-0.18	-0.18	1.70	1.77
1991	74.93	74.42	75.11	74.60	73.74	72.93	-0.18	-0.18	1.37	1.67
1992	75.35	74.80	75.50	74.95	74.41	73.63	-0.15	-0.15	1.09	1.32
1993	75.47	74.87	75.61	75.02	74.85	73.85	-0.15	-0.15	0.76	1.17
1994	76.06	75.42	76.23	75.60	74.92	73.92	-0.17	-0.17	1.31	1.67
1995	76.17	75.51	76.33	75.67	75.52	74.52	-0.16	-0.16	0.80	1.15
1996	76.51	75.82	76.65	75.96	75.63	74.91	-0.14	-0.14	1.02	1.05
1997	76.69	76.00	76.83	76.14	75.94	74.94	-0.14	-0.14	0.89	1.20
1998	76.81	76.12	76.96	76.27	75.79	75.09	-0.15	-0.15	1.17	1.18
1999	76.99	76.31	77.14	76.45	76.34	75.34	-0.14	-0.15	0.80	1.11
2000	77.34	76.66	77.51	76.83	76.57	75.57	-0.17	-0.17	0.94	1.25
2001	77.51	76.82	77.63	76.94	77.00	76.00	-0.12	-0.12	0.63	0.95
2002	77.69	76.97	77.78	77.06	77.38	76.38	-0.10	-0.10	0.40	0.68
2003	77.88	77.17	77.98	77.27	77.34	76.58	-0.10	-0.10	0.64	0.68
2004	78.13	77.39	78.29	77.55	77.28	76.28	-0.16	-0.16	1.01	1.28
2005	78.39	77.59	78.53	77.73	77.32	76.58	-0.13	-0.13	1.20	1.14
2006	78.66	77.90	78.77	78.02	78.04	77.04	-0.12	-0.12	0.74	0.98
2007	78.90	78.11	78.99	78.21	78.34	77.34	-0.09	-0.09	0.65	0.86
2008	79.06	78.26	79.17	78.37	78.53	77.53	-0.11	-0.11	0.64	0.84
2009	79.30	78.50	79.39	78.59	78.98	77.98	-0.09	-0.09	0.41	0.61
2010	79.48	78.69	79.55	78.76	79.27	78.27	-0.07	-0.07	0.28	0.49
2011	79.77	78.94	79.80	78.98	79.49	78.49	-0.03	-0.03	0.31	0.49
2012	79.84	79.07	79.90	79.13	79.53	78.53	-0.06	-0.06	0.37	0.60
2013	80.05	79.28	80.13	79.36	79.53	78.78	-0.08	-0.08	0.59	0.58
2014	80.31	79.51	80.32	79.53	80.08	79.25	-0.01	-0.02	0.25	0.28
2015	80.29	79.51	80.31	79.53	80.14	79.28	-0.01	-0.01	0.17	0.24
2016	80.53	79.74	80.52	79.73	80.41	79.57	0.01	0.01	0.11	0.17
2017	80.69	79.88	80.68	79.88	80.59	79.72	0.00	0.00	0.09	0.15
2018	80.77	79.95	80.76	79.94	80.77	79.77	0.01	0.01	0.00	0.17
2019	81.35	80.53	81.30	80.48	81.38	80.54	0.05	0.05	-0.08	-0.07

Notes: PLE0 (life expectancy at birth); PLE1 (life expectancy at age 1); a-b = total population minus native-born; b-c = native-born minus foreign-born

Source: authors' calculations based upon the Swedish register data "Ageing Well"

Table 8. Robustness check of life expectancy at birth (PLE0) versus life expectancy at age 1 (PLE1), women, Sweden.

Year	Total population (a)		Native-born (b)		Foreign-born (c)		(a-b)		(b-c)	
	e0	e1	e0	e1	e0	e1	e0	e1	e0	e1
1990	80.39	79.83	80.43	79.87	79.87	79.78	-0.04	-0.04	0.56	0.09
1991	80.54	80.00	80.59	80.05	79.87	79.67	-0.05	-0.05	0.72	0.38
1992	80.78	80.16	80.82	80.20	80.91	79.91	-0.04	-0.04	-0.09	0.29
1993	80.76	80.10	80.81	80.15	80.49	79.76	-0.05	-0.05	0.32	0.38
1994	81.38	80.70	81.37	80.69	81.16	80.91	0.01	0.01	0.20	-0.22
1995	81.45	80.73	81.51	80.79	81.41	80.41	-0.06	-0.06	0.10	0.38
1996	81.52	80.82	81.55	80.85	81.22	80.84	-0.03	-0.03	0.33	0.01
1997	81.79	81.05	81.87	81.12	81.20	80.53	-0.07	-0.07	0.66	0.59
1998	81.89	81.14	81.97	81.22	81.43	80.74	-0.09	-0.09	0.55	0.49
1999	81.86	81.07	81.93	81.15	81.69	80.69	-0.07	-0.07	0.24	0.46
2000	82.01	81.24	82.04	81.27	81.30	81.17	-0.03	-0.03	0.74	0.10
2001	82.05	81.32	82.07	81.34	82.42	81.42	-0.02	-0.02	-0.35	-0.08
2002	82.08	81.33	82.13	81.39	82.00	81.00	-0.05	-0.05	0.13	0.39
2003	82.41	81.62	82.40	81.62	82.28	81.81	0.00	0.00	0.13	-0.19
2004	82.44	81.68	82.51	81.75	82.36	81.36	-0.07	-0.07	0.15	0.39
2005	82.75	81.95	82.77	81.96	81.81	81.92	-0.02	-0.01	0.96	0.04
2006	82.89	82.11	82.90	82.12	83.16	82.16	-0.01	-0.01	-0.26	-0.04
2007	82.93	82.12	82.96	82.16	82.71	81.99	-0.03	-0.03	0.26	0.17
2008	83.10	82.31	83.15	82.36	83.07	82.07	-0.05	-0.05	0.08	0.29
2009	83.32	82.52	83.35	82.55	83.02	82.29	-0.03	-0.03	0.34	0.27
2010	83.46	82.67	83.43	82.63	83.70	82.98	0.03	0.03	-0.27	-0.35
2011	83.65	82.81	83.66	82.82	83.63	82.63	-0.01	-0.01	0.03	0.19
2012	83.52	82.71	83.47	82.67	83.88	82.88	0.05	0.05	-0.41	-0.22
2013	83.70	82.91	83.69	82.90	83.60	82.87	0.00	0.00	0.09	0.03
2014	84.04	83.19	84.02	83.17	84.17	83.17	0.02	0.02	-0.15	0.00
2015	84.00	83.19	83.99	83.17	83.59	83.07	0.01	0.02	0.40	0.10
2016	84.07	83.27	84.06	83.26	84.21	83.21	0.01	0.01	-0.15	0.05
2017	84.10	83.29	84.07	83.27	84.30	83.30	0.03	0.03	-0.23	-0.04
2018	84.24	83.39	84.19	83.35	83.99	83.44	0.04	0.04	0.20	-0.09
2019	84.74	83.90	84.68	83.83	85.02	84.02	0.06	0.06	-0.34	-0.19

Notes: PLE0 (life expectancy at birth); PLE1 (life expectancy at age 1); a-b = total population minus native-born; b-c = native-born minus foreign-born

Source: authors' calculations based upon the Swedish register data "Ageing Well"

Supplementary file 3

Table 1. Comparison of period life expectancy at birth in four Nordic countries with the Human Mortality Database, men, 1990-2019.

Year	Denmark			Finland			Norway			Sweden		
	HMD	Est.	**Diff.**	HMD	Est.	**Diff.**	HMD	Est.	**Diff.**	HMD	Est.	**Diff.**
1990	72.02	71.99	**-0.03**	70.94	71.14	**0.20**	73.45	73.44	**-0.01**	74.81	74.78	**-0.03**
1991	72.47	72.43	**-0.04**	71.33	71.59	**0.26**	74.02	74.01	**-0.01**	74.95	74.93	**-0.02**
1992	72.56	72.54	**-0.02**	71.67	71.89	**0.22**	74.17	74.16	**-0.01**	75.36	75.35	**-0.01**
1993	72.60	72.57	**-0.03**	72.11	72.25	**0.14**	74.24	74.23	**-0.01**	75.49	75.47	**-0.02**
1994	72.78	72.75	**-0.03**	72.80	73.01	**0.21**	74.89	74.89	**0.00**	76.08	76.06	**-0.02**
1995	72.73	72.70	**-0.03**	72.81	72.91	**0.10**	74.80	74.79	**-0.01**	76.18	76.17	**-0.01**
1996	73.05	73.03	**-0.02**	73.03	73.16	**0.13**	75.37	75.36	**-0.01**	76.52	76.51	**-0.01**
1997	73.56	73.56	**0.00**	73.43	73.57	**0.14**	75.46	75.45	**-0.01**	76.70	76.69	**-0.01**
1998	73.94	73.94	**0.00**	73.52	73.68	**0.16**	75.53	75.50	**-0.03**	76.87	76.81	**-0.06**
1999	74.21	74.19	**-0.02**	73.74	73.84	**0.10**	75.61	75.61	**0.00**	77.07	77.00	**-0.07**
2000	74.44	74.43	**-0.01**	74.16	74.29	**0.13**	75.96	75.95	**-0.01**	77.38	77.35	**-0.03**
2001	74.67	74.66	**-0.01**	74.58	74.69	**0.11**	76.21	76.19	**-0.02**	77.54	77.51	**-0.03**
2002	74.80	74.79	**-0.01**	74.87	74.91	**0.04**	76.40	76.39	**-0.01**	77.71	77.69	**-0.02**
2003	75.15	75.00	**-0.15**	75.13	75.20	**0.07**	77.04	77.04	**0.00**	77.91	77.88	**-0.03**
2004	75.29	75.36	**0.07**	75.31	75.35	**0.04**	77.51	77.50	**-0.02**	78.35	78.32	**-0.03**
2005	75.94	75.91	**-0.03**	75.53	75.72	**0.19**	77.72	77.72	**0.00**	78.42	78.39	**-0.03**
2006	75.90	75.89	**-0.01**	75.82	75.90	**0.08**	78.12	78.11	**-0.01**	78.69	78.66	**-0.03**
2007	76.13	76.12	**-0.01**	75.87	75.95	**0.08**	78.24	78.23	**-0.01**	78.93	78.90	**-0.03**
2008	76.48	76.47	**-0.01**	76.32	76.42	**0.10**	78.32	78.31	**-0.01**	79.09	79.06	**-0.03**
2009	76.84	76.83	**-0.01**	76.48	76.55	**0.07**	78.59	78.59	**0.00**	79.34	79.30	**-0.04**
2010	77.12	77.10	**-0.02**	76.72	76.76	**0.04**	78.85	78.85	**0.00**	79.52	79.48	**-0.04**
2011	77.70	77.69	**-0.01**	77.19	77.21	**0.02**	79.00	78.99	**-0.01**	79.80	79.77	**-0.03**
2012	78.07	78.04	**-0.03**	77.50	77.52	**0.02**	79.42	79.41	**-0.01**	79.87	79.84	**-0.03**
2013	78.27	78.24	**-0.03**	77.88	77.86	**-0.02**	79.66	79.65	**-0.01**	80.10	80.05	**-0.05**
2014	78.57	78.56	**-0.01**	78.13	78.14	**0.01**	80.03	80.02	**-0.01**	80.36	80.31	**-0.05**
2015	78.77	78.75	**-0.02**	78.59	78.56	**-0.03**	80.35	80.36	**0.01**	80.32	80.30	**-0.02**
2016	78.95	78.93	**-0.02**	78.43	78.41	**-0.02**	80.60	80.60	**0.00**	80.57	80.53	**-0.04**
2017	79.09	79.08	**-0.01**	78.72	78.72	**0.00**	80.91	80.91	**0.00**	80.73	80.73	**0.00**
2018	79.02	79.00	**-0.02**	78.91	78.91	**0.00**	81.00	81.00	**0.00**	80.79	80.79	**0.00**
2019	79.44	79.43	**-0.01**	79.22	79.23	**0.01**	81.19	81.19	**0.00**	81.35	81.34	**-0.01**

Notes: HMD (Human Mortality Database) Est. (our estimations based upon the register data); Diff. (difference, Est. minus HMD)

Source: authors' calculations based upon respective register data for each country; Human Mortality Database

Table 2. Comparison of period life expectancy at birth in four Nordic countries with the Human Mortality Database, women, 1990-2019.

Year	Denmark			Finland			Norway			Sweden		
	HMD	Est.	**Diff.**	HMD	Est.	**Diff.**	HMD	Est.	**Diff.**	HMD	Est.	**Diff.**
1990	77.73	77.70	**-0.03**	78.88	79.12	**0.24**	79.80	79.80	**0.00**	80.40	80.38	**-0.02**
1991	77.98	77.96	**-0.02**	79.32	79.52	**0.20**	80.09	80.10	**0.01**	80.54	80.53	**-0.01**
1992	77.97	77.93	**-0.04**	79.44	79.57	**0.13**	80.35	80.36	**0.01**	80.78	80.78	**0.00**
1993	77.77	77.75	**-0.02**	79.48	79.54	**0.06**	80.24	80.24	**0.00**	80.78	80.76	**-0.02**
1994	78.10	78.08	**-0.02**	80.15	80.31	**0.16**	80.65	80.65	**0.00**	81.38	81.38	**0.00**
1995	77.84	77.81	**-0.03**	80.21	80.27	**0.06**	80.81	80.82	**0.01**	81.44	81.45	**0.01**
1996	78.26	78.22	**-0.04**	80.55	80.61	**0.06**	81.06	81.06	**0.00**	81.52	81.52	**0.00**
1997	78.47	78.46	**-0.01**	80.51	80.60	**0.09**	80.97	80.97	**0.00**	81.80	81.79	**-0.01**
1998	78.88	78.88	**0.00**	80.84	80.94	**0.10**	81.26	81.25	**-0.01**	81.91	81.89	**-0.02**
1999	78.89	78.88	**-0.01**	81.03	81.14	**0.11**	81.12	81.12	**0.00**	81.89	81.85	**-0.04**
2000	79.12	79.11	**-0.01**	81.02	81.09	**0.07**	81.37	81.38	**0.01**	82.02	82.01	**-0.02**
2001	79.21	79.21	**0.00**	81.54	81.54	**0.00**	81.52	81.52	**0.00**	82.05	82.05	**0.00**
2002	79.34	79.32	**-0.02**	81.53	81.58	**0.05**	81.46	81.46	**0.00**	82.08	82.08	**0.00**
2003	79.80	79.72	**-0.08**	81.81	81.87	**0.06**	81.93	81.93	**0.00**	82.41	82.40	**-0.01**
2004	80.05	80.07	**0.02**	82.27	82.18	**-0.09**	82.33	82.34	**0.01**	82.66	82.65	**-0.01**
2005	80.45	80.43	**-0.02**	82.30	82.51	**0.21**	82.51	82.52	**0.01**	82.75	82.75	**0.00**
2006	80.51	80.51	**0.00**	82.83	82.88	**0.05**	82.66	82.66	**0.00**	82.90	82.89	**-0.01**
2007	80.53	80.51	**-0.02**	82.86	82.88	**0.02**	82.67	82.66	**-0.01**	82.94	82.93	**-0.01**
2008	80.92	80.91	**-0.01**	83.01	83.03	**0.02**	82.96	82.95	**-0.01**	83.12	83.10	**-0.02**
2009	81.03	81.02	**-0.01**	83.11	83.18	**0.07**	83.06	83.05	**-0.01**	83.33	83.32	**-0.01**
2010	81.33	81.32	**-0.01**	83.24	83.21	**-0.03**	83.15	83.15	**0.00**	83.47	83.46	**-0.01**
2011	81.83	81.81	**-0.02**	83.54	83.56	**0.02**	83.44	83.45	**0.01**	83.67	83.65	**-0.02**
2012	82.04	82.02	**-0.02**	83.41	83.40	**-0.01**	83.42	83.40	**-0.02**	83.53	83.51	**-0.02**
2013	82.31	82.29	**-0.02**	83.82	83.76	**-0.06**	83.61	83.60	**-0.01**	83.72	83.69	**-0.03**
2014	82.67	82.67	**0.00**	83.85	83.83	**-0.02**	84.10	84.10	**0.00**	84.05	84.03	**-0.02**
2015	82.69	82.68	**-0.01**	84.17	84.13	**-0.04**	84.12	84.15	**0.03**	84.02	84.00	**-0.02**
2016	82.79	82.77	**-0.02**	84.12	84.09	**-0.03**	84.15	84.17	**0.02**	84.08	84.07	**-0.01**
2017	83.12	83.11	**-0.01**	84.23	84.22	**-0.01**	84.26	84.28	**0.02**	84.12	84.11	**-0.01**
2018	82.96	82.94	**-0.02**	84.31	84.30	**-0.01**	84.47	84.49	**0.02**	84.26	84.26	**0.00**
2019	83.42	83.40	**-0.02**	84.56	84.56	**0.00**	84.68	84.68	**0.00**	84.73	84.73	**0.00**

Notes:
HMD (Human Mortality Database) Est. (our estimations based upon the register data); Diff. (difference, Est. minus HMD)

Source: authors' calculations based upon respective register data for each country; Human Mortality Database

Supplementary file 4

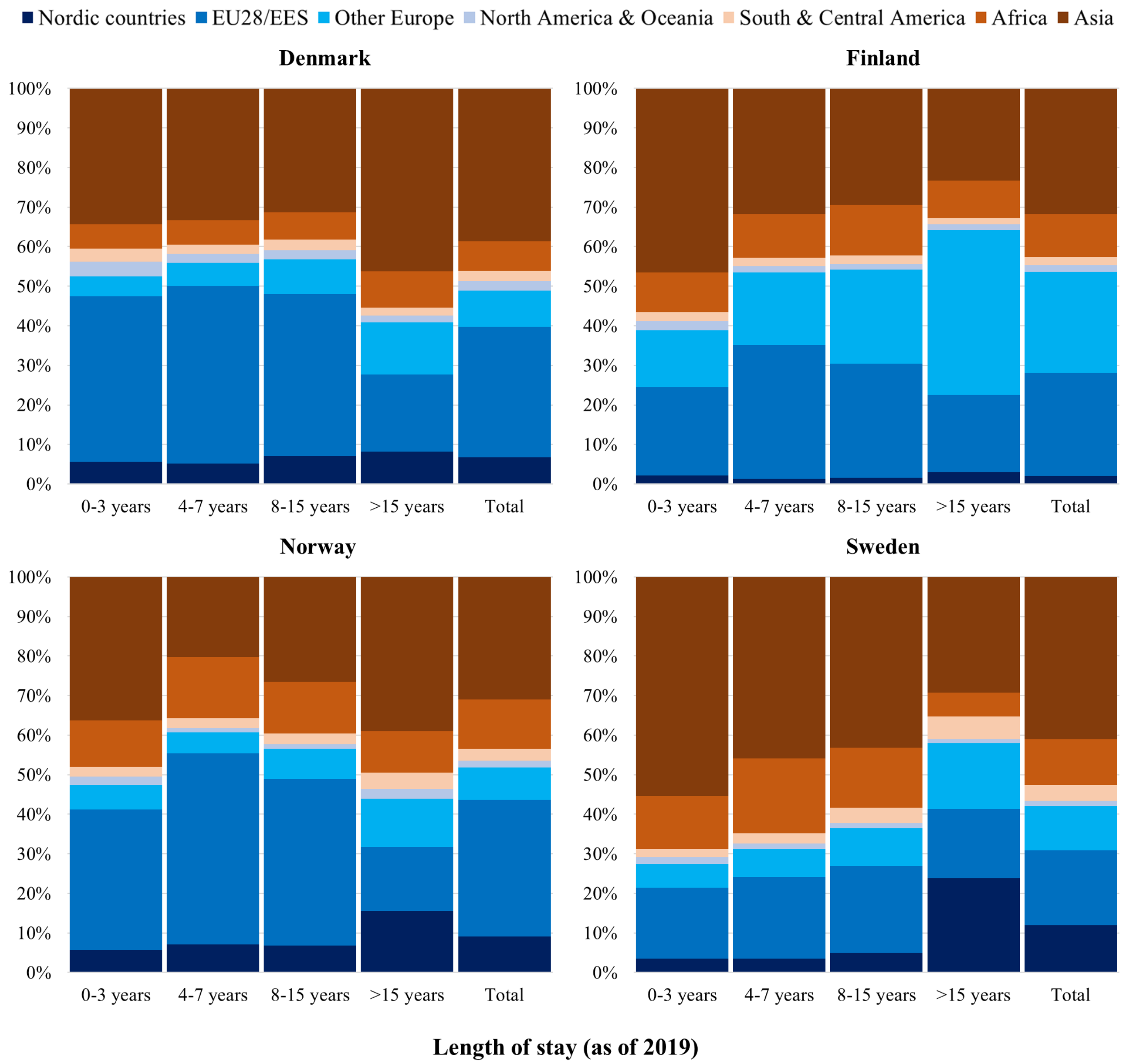

Figure 1. Length of stay by origin composition of migrants in Denmark, Finland, Norway and Sweden in 2019.

Notes: Asia includes Turkey and the Middle East; South Central America includes Mexico and Caribbean

Source: derived from Nordic Statistics Database table MIGR06: Foreign-born with two foreign-born parents by reporting country, country background, time, sex and length of stay (https://www.nordicstatistics.org/population)

Supplementary file 5

Table 1. Period life expectancy at age 25 among men in four Nordic countries, men, 1990-2019.

Year	Denmark, e25				Finland, e25				Norway, e25				Sweden, e25			
	Total	NB	FB	**Total - NB**	Total	NB	FB	**Total - NB**	Total	NB	FB	**Total - NB**	Total	NB	FB	**Total - NB**
1990	48.32	48.33	47.91	**-0.02**	47.16	47.14	49.46	**0.02**	49.82	49.78	50.51	**0.03**	50.92	51.10	49.49	**-0.17**
1991	48.75	48.75	48.86	**0.01**	47.52	47.48	51.35	**0.03**	50.19	50.17	50.51	**0.02**	50.98	51.14	49.59	**-0.17**
1992	48.76	48.75	48.98	**0.01**	47.71	47.69	49.36	**0.02**	50.39	50.35	51.30	**0.05**	51.31	51.46	50.15	**-0.15**
1993	48.62	48.61	49.11	**0.01**	48.00	47.95	52.21	**0.05**	50.30	50.24	51.32	**0.06**	51.41	51.56	50.30	**-0.15**
1994	48.83	48.83	49.06	**0.00**	48.81	48.79	50.92	**0.02**	50.91	50.87	51.62	**0.05**	51.89	52.06	50.62	**-0.16**
1995	48.76	48.74	49.25	**0.02**	48.66	48.63	50.73	**0.03**	50.84	50.81	51.74	**0.03**	51.98	52.13	50.92	**-0.16**
1996	49.10	49.08	50.13	**0.02**	48.89	48.86	50.52	**0.02**	51.30	51.27	51.48	**0.02**	52.24	52.38	51.40	**-0.14**
1997	49.56	49.52	50.72	**0.04**	49.29	49.23	53.82	**0.06**	51.43	51.42	51.41	**0.02**	52.44	52.58	51.53	**-0.14**
1998	49.83	49.81	50.41	**0.02**	49.31	49.25	53.14	**0.06**	51.48	51.46	51.75	**0.02**	52.63	52.75	51.81	**-0.12**
1999	50.13	50.11	50.58	**0.03**	49.56	49.51	53.02	**0.05**	51.65	51.62	52.20	**0.03**	52.76	52.89	51.97	**-0.13**
2000	50.46	50.42	50.87	**0.04**	50.00	49.95	52.95	**0.05**	51.97	51.96	52.15	**0.01**	53.14	53.30	52.11	**-0.16**
2001	50.56	50.55	50.92	**0.01**	50.43	50.40	52.07	**0.03**	52.22	52.19	52.59	**0.03**	53.30	53.40	52.67	**-0.11**
2002	50.69	50.65	51.59	**0.04**	50.61	50.57	52.21	**0.04**	52.31	52.26	53.05	**0.05**	53.45	53.55	52.82	**-0.10**
2003	50.90	50.85	51.70	**0.05**	50.90	50.85	53.75	**0.06**	52.95	52.85	54.50	**0.10**	53.64	53.74	53.08	**-0.10**
2004	51.23	51.18	52.19	**0.05**	51.09	51.02	54.90	**0.08**	53.37	53.31	53.77	**0.06**	53.96	54.11	52.92	**-0.15**
2005	51.77	51.70	53.05	**0.07**	51.35	51.28	54.46	**0.07**	53.58	53.54	53.99	**0.04**	54.05	54.17	53.20	**-0.12**
2006	51.70	51.68	51.96	**0.02**	51.59	51.53	54.12	**0.06**	53.94	53.90	54.31	**0.04**	54.36	54.48	53.61	**-0.12**
2007	51.93	51.86	53.25	**0.06**	51.60	51.51	53.70	**0.09**	53.95	53.89	54.52	**0.06**	54.55	54.65	53.87	**-0.10**
2008	52.33	52.26	53.49	**0.07**	52.09	51.98	55.17	**0.10**	54.11	54.05	54.51	**0.05**	54.69	54.80	53.90	**-0.11**
2009	52.43	52.40	52.92	**0.03**	52.14	52.04	55.00	**0.10**	54.38	54.27	55.64	**0.10**	54.96	55.04	54.49	**-0.08**
2010	52.73	52.63	53.95	**0.09**	52.39	52.31	54.70	**0.08**	54.54	54.47	55.13	**0.07**	55.09	55.17	54.56	**-0.08**
2011	53.30	53.22	54.40	**0.08**	52.80	52.72	54.85	**0.08**	54.73	54.60	56.10	**0.12**	55.35	55.38	54.93	**-0.04**
2012	53.63	53.59	54.01	**0.04**	53.06	52.95	55.37	**0.11**	55.02	54.90	55.74	**0.11**	55.45	55.52	54.90	**-0.07**
2013	53.79	53.71	54.84	**0.08**	53.33	53.23	55.60	**0.11**	55.19	55.03	56.29	**0.15**	55.66	55.73	55.23	**-0.07**
2014	54.18	54.11	55.00	**0.07**	53.63	53.53	56.50	**0.10**	55.60	55.48	56.33	**0.12**	55.88	55.90	55.67	**-0.01**
2015	54.34	54.25	55.52	**0.09**	54.02	53.93	55.71	**0.10**	55.90	55.71	57.68	**0.19**	55.90	55.91	55.65	**-0.01**
2016	54.50	54.43	55.19	**0.08**	53.87	53.71	57.93	**0.16**	56.10	55.96	56.99	**0.14**	56.07	56.06	55.92	**0.02**
2017	54.73	54.70	55.36	**0.03**	54.24	54.10	57.12	**0.14**	56.48	56.35	57.58	**0.13**	56.29	56.25	56.32	**0.04**
2018	54.61	54.53	55.14	**0.08**	54.40	54.29	56.87	**0.11**	56.52	56.39	57.70	**0.13**	56.36	56.31	56.32	**0.04**
2019	55.00	54.91	55.83	**0.09**	54.77	54.64	56.68	**0.13**	56.78	56.66	57.51	**0.12**	56.88	56.84	56.32	**0.04**

Notes: PLE (Period Life Expectancy); Total (total resident population); NB (native-born population); FB (foreign-born population)

Source: authors' calculations based upon respective register data for each country

Table 2. Period life expectancy at age 50 among men in four Nordic countries, men, 1990-2019.

**Year**	**Denmark, e50**				**Finland, e50**				**Norway, e50**				**Sweden, e50**			
	Total	NB	FB	**Total - NB**	Total	NB	FB	**Total - NB**	Total	NB	FB	**Total - NB**	Total	NB	FB	**Total - NB**
1990	25.34	25.36	24.88	**-0.02**	24.84	24.83	26.00	**0.01**	26.55	26.55	26.78	**0.00**	27.50	27.60	26.53	**-0.10**
1991	25.77	25.77	25.88	**0.00**	25.28	25.27	27.38	**0.01**	26.82	26.82	26.94	**0.01**	27.59	27.69	26.68	**-0.09**
1992	25.73	25.72	25.96	**0.01**	25.28	25.28	25.90	**0.00**	27.01	27.00	27.52	**0.02**	27.82	27.92	26.91	**-0.11**
1993	25.64	25.61	26.37	**0.02**	25.47	25.45	28.21	**0.02**	26.91	26.90	27.40	**0.02**	27.89	27.99	27.16	**-0.09**
1994	25.87	25.86	26.28	**0.01**	26.22	26.21	27.58	**0.01**	27.45	27.43	27.80	**0.01**	28.41	28.53	27.38	**-0.12**
1995	25.75	25.74	26.04	**0.01**	26.04	26.03	27.21	**0.01**	27.39	27.38	28.05	**0.01**	28.40	28.50	27.67	**-0.10**
1996	26.03	26.00	27.06	**0.03**	26.34	26.34	27.12	**0.01**	27.83	27.81	27.90	**0.01**	28.60	28.69	28.09	**-0.09**
1997	26.44	26.41	27.52	**0.03**	26.65	26.62	29.79	**0.03**	27.95	27.95	27.63	**-0.01**	28.76	28.86	28.00	**-0.11**
1998	26.65	26.64	27.09	**0.01**	26.62	26.59	29.10	**0.03**	28.08	28.08	28.23	**0.00**	28.89	29.00	28.15	**-0.11**
1999	26.87	26.87	27.01	**0.00**	26.86	26.83	29.49	**0.03**	28.15	28.13	28.61	**0.02**	29.06	29.15	28.52	**-0.09**
2000	27.16	27.15	27.22	**0.00**	27.26	27.24	29.14	**0.02**	28.65	28.66	28.53	**-0.02**	29.39	29.49	28.71	**-0.10**
2001	27.23	27.23	27.56	**0.01**	27.62	27.61	28.57	**0.01**	28.80	28.79	28.99	**0.01**	29.58	29.68	28.94	**-0.11**
2002	27.37	27.35	28.11	**0.02**	27.68	27.67	28.53	**0.01**	28.82	28.81	29.19	**0.01**	29.62	29.73	28.92	**-0.11**
2003	27.56	27.53	28.13	**0.02**	27.94	27.92	30.02	**0.03**	29.41	29.37	30.44	**0.03**	29.82	29.92	29.23	**-0.11**
2004	27.88	27.86	28.64	**0.02**	28.24	28.21	31.27	**0.03**	29.74	29.73	29.74	**0.01**	30.16	30.29	29.22	**-0.13**
2005	28.26	28.21	29.45	**0.05**	28.41	28.38	30.68	**0.03**	29.93	29.92	30.11	**0.01**	30.20	30.32	29.36	**-0.12**
2006	28.22	28.21	28.35	**0.01**	28.57	28.54	30.41	**0.03**	30.27	30.26	30.45	**0.01**	30.43	30.56	29.63	**-0.13**
2007	28.44	28.40	29.62	**0.05**	28.67	28.64	29.76	**0.02**	30.20	30.17	30.64	**0.04**	30.67	30.79	29.92	**-0.12**
2008	28.75	28.69	29.89	**0.06**	29.08	29.04	31.35	**0.05**	30.36	30.35	30.54	**0.02**	30.81	30.94	29.92	**-0.13**
2009	28.83	28.81	29.28	**0.02**	29.14	29.11	31.21	**0.04**	30.66	30.60	31.75	**0.06**	31.06	31.19	30.43	**-0.13**
2010	29.05	29.00	29.99	**0.05**	29.23	29.20	30.95	**0.03**	30.76	30.74	31.16	**0.02**	31.15	31.25	30.56	**-0.10**
2011	29.59	29.55	30.48	**0.04**	29.55	29.51	31.13	**0.03**	31.00	30.96	32.05	**0.04**	31.39	31.48	30.80	**-0.09**
2012	29.79	29.76	30.04	**0.02**	29.78	29.74	31.40	**0.04**	31.15	31.12	31.61	**0.03**	31.49	31.61	30.75	**-0.12**
2013	29.93	29.88	30.82	**0.05**	30.04	30.00	31.75	**0.04**	31.34	31.31	32.07	**0.03**	31.73	31.84	31.18	**-0.11**
2014	30.35	30.31	31.01	**0.04**	30.25	30.19	32.79	**0.05**	31.73	31.71	32.18	**0.01**	31.96	32.03	31.57	**-0.07**
2015	30.40	30.33	31.45	**0.07**	30.49	30.46	31.61	**0.03**	31.93	31.85	33.40	**0.08**	31.96	32.03	31.54	**-0.07**
2016	30.60	30.55	31.18	**0.06**	30.39	30.34	33.71	**0.05**	32.17	32.14	32.78	**0.03**	32.16	32.23	31.77	**-0.06**
2017	30.75	30.72	31.41	**0.03**	30.72	30.65	33.04	**0.06**	32.40	32.35	33.27	**0.05**	32.38	32.42	32.18	**-0.04**
2018	30.64	30.59	31.10	**0.05**	30.84	30.79	32.92	**0.05**	32.56	32.51	33.54	**0.05**	32.38	32.42	32.18	**-0.04**
2019	30.98	30.92	31.68	**0.05**	31.24	31.18	32.62	**0.05**	32.78	32.74	33.32	**0.04**	32.85	32.89	32.18	**-0.04**

Notes: PLE (Period Life Expectancy); Total (total resident population); NB (native-born population); FB (foreign-born population)

Source: authors' calculations based upon respective register data for each country

Table 3. Period life expectancy at age 75 among men in four Nordic countries, men, 1990-2019.

Year	Denmark, e75				Finland, e75				Norway, e75				Sweden, e75			
	Total	NB	FB	**Total - NB**	Total	NB	FB	**Total - NB**	Total	NB	FB	**Total - NB**	Total	NB	FB	**Total - NB**
1990	8.43	8.43	8.52	**0.00**	8.37	8.37	8.48	**0.00**	8.66	8.65	8.89	**0.00**	8.94	8.94	8.92	**0.00**
1991	8.68	8.69	8.45	**0.00**	8.59	8.59	8.60	**0.00**	8.79	8.80	8.59	**0.00**	9.03	9.03	9.03	**0.00**
1992	8.60	8.60	8.64	**0.00**	8.45	8.45	7.93	**-0.01**	8.83	8.82	9.09	**0.00**	9.17	9.16	9.40	**0.01**
1993	8.39	8.38	8.72	**0.01**	8.42	8.41	9.28	**0.01**	8.67	8.67	8.52	**0.00**	9.06	9.04	9.38	**0.01**
1994	8.62	8.62	8.59	**0.00**	8.85	8.85	9.26	**0.00**	9.10	9.10	9.38	**0.00**	9.47	9.47	9.45	**0.00**
1995	8.45	8.45	8.60	**0.00**	8.85	8.85	9.13	**0.00**	8.85	8.85	9.68	**0.00**	9.37	9.36	9.66	**0.01**
1996	8.61	8.58	9.72	**0.02**	8.87	8.88	8.18	**-0.01**	9.14	9.15	8.65	**-0.01**	9.46	9.45	9.82	**0.01**
1997	8.78	8.76	9.68	**0.02**	9.06	9.06	9.46	**0.00**	9.11	9.11	9.03	**0.00**	9.58	9.58	9.64	**0.00**
1998	8.87	8.87	9.11	**0.01**	9.04	9.03	9.96	**0.01**	9.23	9.22	9.54	**0.01**	9.61	9.61	9.73	**0.00**
1999	8.90	8.89	9.10	**0.00**	9.19	9.17	10.85	**0.02**	9.14	9.13	9.58	**0.01**	9.66	9.65	9.86	**0.00**
2000	9.04	9.04	8.99	**0.00**	9.27	9.26	10.65	**0.01**	9.41	9.40	9.88	**0.01**	9.84	9.85	9.96	**0.00**
2001	9.05	9.04	9.20	**0.00**	9.43	9.43	9.73	**0.00**	9.41	9.41	9.30	**0.00**	9.95	9.95	10.20	**0.00**
2002	9.15	9.13	10.03	**0.02**	9.51	9.52	8.89	**-0.01**	9.49	9.47	10.29	**0.02**	9.95	9.95	10.04	**-0.01**
2003	9.21	9.20	9.69	**0.01**	9.74	9.73	10.27	**0.01**	9.89	9.89	10.33	**0.00**	10.08	10.09	10.27	**-0.01**
2004	9.41	9.40	10.04	**0.01**	10.10	10.09	11.67	**0.02**	10.15	10.16	9.62	**-0.01**	10.39	10.41	10.18	**-0.02**
2005	9.59	9.58	9.99	**0.01**	10.24	10.23	11.48	**0.01**	10.11	10.10	10.29	**0.01**	10.31	10.34	10.01	**-0.04**
2006	9.69	9.68	10.04	**0.01**	10.26	10.25	11.11	**0.01**	10.43	10.42	10.96	**0.01**	10.48	10.49	10.45	**-0.01**
2007	9.83	9.80	11.06	**0.03**	10.34	10.34	10.41	**0.00**	10.25	10.25	10.30	**0.00**	10.59	10.61	10.48	**-0.02**
2008	9.94	9.93	10.25	**0.01**	10.70	10.70	10.74	**0.00**	10.35	10.35	10.36	**0.00**	10.69	10.70	10.57	**-0.01**
2009	9.94	9.92	10.40	**0.01**	10.58	10.57	11.67	**0.01**	10.64	10.64	11.06	**0.01**	10.79	10.80	10.99	**0.00**
2010	10.14	10.13	10.74	**0.02**	10.60	10.59	11.47	**0.01**	10.69	10.68	11.00	**0.00**	10.84	10.85	10.81	**-0.01**
2011	10.38	10.36	11.15	**0.02**	10.78	10.78	10.97	**0.00**	10.80	10.78	11.78	**0.03**	11.00	11.02	10.85	**-0.02**
2012	10.51	10.50	10.65	**0.00**	10.85	10.85	11.14	**0.00**	10.88	10.87	11.11	**0.01**	10.97	10.99	10.87	**-0.02**
2013	10.59	10.58	10.91	**0.01**	11.05	11.05	11.30	**0.00**	11.05	11.04	11.75	**0.02**	11.22	11.26	11.14	**-0.04**
2014	10.95	10.94	11.33	**0.01**	11.09	11.08	12.42	**0.01**	11.26	11.25	11.84	**0.01**	11.36	11.38	11.33	**-0.02**
2015	10.91	10.89	11.32	**0.01**	11.19	11.19	11.49	**0.00**	11.33	11.31	12.30	**0.02**	11.35	11.37	11.29	**-0.01**
2016	11.05	11.05	11.14	**0.01**	11.17	11.15	13.64	**0.02**	11.58	11.57	12.15	**0.01**	11.50	11.51	11.43	**-0.01**
2017	11.05	11.03	11.75	**0.02**	11.25	11.24	12.20	**0.01**	11.60	11.59	12.31	**0.01**	11.54	11.55	11.55	**-0.01**
2018	10.90	10.90	10.92	**0.00**	11.37	11.36	12.51	**0.01**	11.70	11.69	12.50	**0.01**	11.59	11.60	11.55	**-0.01**
2019	11.26	11.25	11.61	**0.01**	11.58	11.58	11.70	**0.00**	11.89	11.89	12.22	**0.00**	11.92	11.92	11.56	**-0.01**

Notes: PLE (Period Life Expectancy); Total (total resident population); NB (native-born population); FB (foreign-born population)

Source: authors' calculations based upon respective register data for each country

Table 4. Period life expectancy at age 25 among women in four Nordic countries, men, 1990-2019.

Year	Denmark, e25				Finland, e25				Norway, e25				Sweden, e25			
	Total	NB	FB	**Total - NB**	Total	NB	FB	**Total - NB**	Total	NB	FB	**Total - NB**	Total	NB	FB	**Total - NB**
1990	53.65	53.64	53.83	**0.00**	54.66	54.65	54.21	**0.00**	55.66	55.66	55.86	**0.00**	56.18	56.21	56.13	**-0.03**
1991	53.86	53.87	53.52	**-0.01**	54.98	54.98	54.82	**0.00**	55.98	55.99	56.34	**-0.01**	56.32	56.36	56.06	**-0.04**
1992	53.77	53.77	53.60	**0.00**	55.06	55.06	55.93	**0.01**	56.14	56.13	56.62	**0.01**	56.47	56.50	56.41	**-0.03**
1993	53.51	53.50	53.74	**0.01**	54.92	54.90	56.13	**0.02**	55.96	55.94	56.37	**0.02**	56.44	56.49	56.11	**-0.05**
1994	53.84	53.82	54.60	**0.02**	55.70	55.69	56.62	**0.01**	56.42	56.43	56.12	**-0.01**	56.96	56.95	57.18	**0.01**
1995	53.52	53.54	53.26	**-0.02**	55.65	55.64	57.51	**0.02**	56.42	56.41	56.95	**0.01**	57.00	57.06	56.67	**-0.06**
1996	53.96	53.96	54.40	**0.00**	56.00	55.99	56.74	**0.01**	56.64	56.63	57.03	**0.01**	57.08	57.12	56.99	**-0.04**
1997	54.14	54.14	54.35	**0.01**	55.98	55.96	57.39	**0.02**	56.63	56.60	57.49	**0.03**	57.36	57.42	56.98	**-0.06**
1998	54.54	54.53	54.62	**0.00**	56.32	56.30	57.47	**0.02**	56.87	56.88	57.09	**-0.01**	57.44	57.51	57.18	**-0.07**
1999	54.43	54.45	54.50	**-0.02**	56.55	56.53	57.70	**0.02**	56.77	56.74	57.27	**0.03**	57.34	57.41	56.95	**-0.07**
2000	54.77	54.72	55.65	**0.05**	56.49	56.48	57.52	**0.01**	57.01	56.97	57.77	**0.04**	57.51	57.54	57.39	**-0.03**
2001	54.89	54.88	55.13	**0.01**	56.85	56.86	56.51	**-0.01**	57.12	57.12	57.31	**0.00**	57.59	57.62	57.60	**-0.03**
2002	54.95	54.90	55.98	**0.05**	56.91	56.90	58.25	**0.01**	57.13	57.09	57.57	**0.04**	57.60	57.65	57.25	**-0.05**
2003	55.30	55.28	55.87	**0.02**	57.19	57.19	57.64	**0.00**	57.54	57.52	57.82	**0.02**	57.90	57.90	57.99	**0.00**
2004	55.72	55.68	56.43	**0.03**	57.63	57.62	58.73	**0.01**	57.93	57.91	58.27	**0.02**	58.06	58.14	57.70	**-0.08**
2005	55.94	55.93	56.17	**0.00**	57.86	57.85	59.07	**0.01**	58.06	57.99	59.18	**0.07**	58.20	58.22	58.10	**-0.02**
2006	56.01	55.96	56.64	**0.05**	58.20	58.17	59.46	**0.03**	58.15	58.13	58.57	**0.02**	58.38	58.40	58.36	**-0.02**
2007	56.03	55.99	56.58	**0.04**	58.28	58.25	59.70	**0.03**	58.18	58.10	59.29	**0.08**	58.38	58.43	58.19	**-0.05**
2008	56.40	56.36	56.98	**0.04**	58.35	58.32	59.29	**0.03**	58.40	58.36	58.97	**0.04**	58.55	58.60	58.33	**-0.05**
2009	56.51	56.47	57.21	**0.04**	58.51	58.49	59.07	**0.01**	58.54	58.47	59.53	**0.07**	58.77	58.81	58.53	**-0.04**
2010	56.79	56.74	57.55	**0.05**	58.55	58.49	60.24	**0.05**	58.64	58.55	59.90	**0.09**	58.91	58.88	59.17	**0.03**
2011	57.32	57.25	58.31	**0.08**	58.86	58.81	59.85	**0.05**	58.90	58.83	60.19	**0.07**	59.06	59.05	59.09	**0.01**
2012	57.46	57.39	58.21	**0.07**	58.76	58.73	59.91	**0.03**	58.82	58.73	60.13	**0.09**	58.93	58.88	59.11	**0.05**
2013	57.76	57.73	58.14	**0.04**	59.05	59.01	60.22	**0.04**	59.05	58.95	60.44	**0.10**	59.12	59.11	59.13	**0.01**
2014	58.13	58.11	58.53	**0.03**	59.10	59.07	59.67	**0.03**	59.46	59.31	61.11	**0.15**	59.41	59.40	59.37	**0.01**
2015	58.14	58.12	58.19	**0.02**	59.36	59.36	59.66	**0.00**	59.50	59.38	60.68	**0.12**	59.42	59.40	59.35	**0.02**
2016	58.23	58.18	58.89	**0.05**	59.37	59.32	61.40	**0.05**	59.56	59.38	61.55	**0.18**	59.50	59.48	59.51	**0.02**
2017	58.53	58.46	59.38	**0.07**	59.52	59.45	60.99	**0.07**	59.66	59.52	61.25	**0.14**	59.52	59.48	59.66	**0.04**
2018	58.34	58.28	58.89	**0.06**	59.57	59.54	60.48	**0.04**	59.84	59.67	61.31	**0.17**	59.68	59.64	59.66	**0.04**
2019	58.79	58.75	59.35	**0.04**	59.89	59.84	61.33	**0.05**	60.05	59.88	61.68	**0.17**	60.12	60.08	59.66	**0.04**

Notes: PLE (Period Life Expectancy); Total (total resident population); NB (native-born population); FB (foreign-born population)

Source: authors' calculations based upon respective register data for each country

Table 5. Period life expectancy at age 50 among women in four Nordic countries, men, 1990-2019.

**Year**	**Denmark, e50**				**Finland, e50**				**Norway, e50**				**Sweden, e50**			
	Total	NB	FB	**Total - NB**	Total	NB	FB	**Total - NB**	Total	NB	FB	**Total - NB**	Total	NB	FB	**Total - NB**
1990	29.92	29.91	30.23	**0.01**	30.77	30.77	30.33	**0.00**	31.69	31.69	32.00	**0.00**	32.19	32.20	32.28	**-0.01**
1991	30.13	30.14	29.75	**-0.01**	31.11	31.12	30.60	**0.00**	31.98	31.98	32.49	**0.00**	32.33	32.35	32.22	**-0.02**
1992	30.03	30.04	29.80	**-0.01**	31.21	31.21	31.61	**0.00**	32.10	32.08	32.61	**0.02**	32.41	32.43	32.40	**-0.02**
1993	29.76	29.75	29.93	**0.01**	31.01	30.99	32.17	**0.01**	31.89	31.88	32.09	**0.01**	32.37	32.38	32.28	**-0.01**
1994	30.10	30.07	30.86	**0.03**	31.77	31.76	32.56	**0.01**	32.34	32.36	31.99	**-0.02**	32.92	32.91	33.18	**0.01**
1995	29.81	29.81	29.83	**0.00**	31.74	31.72	33.32	**0.02**	32.33	32.32	32.89	**0.01**	32.89	32.92	32.71	**-0.03**
1996	30.20	30.17	30.89	**0.03**	32.06	32.06	32.34	**0.00**	32.58	32.58	32.86	**0.00**	32.93	32.96	32.90	**-0.03**
1997	30.35	30.34	30.54	**0.01**	32.11	32.10	33.16	**0.01**	32.54	32.52	33.25	**0.02**	33.17	33.21	32.85	**-0.04**
1998	30.66	30.65	30.77	**0.01**	32.37	32.36	33.35	**0.01**	32.80	32.79	33.27	**0.01**	33.26	33.30	33.13	**-0.04**
1999	30.57	30.55	30.94	**0.01**	32.59	32.58	33.45	**0.01**	32.68	32.66	33.17	**0.02**	33.19	33.25	32.86	**-0.06**
2000	30.83	30.78	31.65	**0.04**	32.62	32.61	33.72	**0.01**	32.92	32.90	33.47	**0.02**	33.29	33.31	33.23	**-0.02**
2001	30.97	30.94	31.32	**0.02**	32.92	32.92	32.54	**0.00**	33.01	33.02	33.09	**-0.01**	33.35	33.37	33.34	**-0.02**
2002	31.00	30.95	32.07	**0.05**	32.94	32.93	34.25	**0.02**	32.95	32.93	33.24	**0.02**	33.35	33.40	32.99	**-0.05**
2003	31.31	31.28	32.02	**0.03**	33.15	33.15	33.67	**0.01**	33.40	33.39	33.61	**0.01**	33.66	33.68	33.69	**-0.02**
2004	31.70	31.67	32.43	**0.04**	33.69	33.67	34.85	**0.02**	33.83	33.81	34.01	**0.02**	33.87	33.93	33.53	**-0.06**
2005	31.87	31.86	32.19	**0.01**	33.86	33.85	35.07	**0.01**	33.93	33.89	34.87	**0.04**	33.91	33.93	33.81	**-0.02**
2006	31.90	31.87	32.35	**0.03**	34.13	34.12	35.14	**0.01**	33.97	33.95	34.36	**0.02**	34.10	34.11	34.08	**-0.01**
2007	31.95	31.92	32.43	**0.03**	34.20	34.17	35.55	**0.02**	33.98	33.93	34.90	**0.05**	34.08	34.13	33.88	**-0.05**
2008	32.31	32.29	32.78	**0.02**	34.26	34.25	34.94	**0.01**	34.18	34.16	34.57	**0.02**	34.24	34.29	34.05	**-0.05**
2009	32.37	32.34	33.08	**0.03**	34.45	34.43	35.04	**0.01**	34.30	34.25	35.15	**0.05**	34.45	34.49	34.19	**-0.04**
2010	32.58	32.55	33.26	**0.03**	34.43	34.41	35.84	**0.03**	34.37	34.33	35.41	**0.04**	34.54	34.52	34.76	**0.02**
2011	33.08	33.03	33.92	**0.05**	34.67	34.66	35.26	**0.02**	34.62	34.56	35.87	**0.06**	34.71	34.73	34.67	**-0.02**
2012	33.20	33.16	33.81	**0.04**	34.58	34.56	35.68	**0.02**	34.48	34.43	35.67	**0.05**	34.59	34.58	34.65	**0.01**
2013	33.45	33.42	33.78	**0.03**	34.86	34.84	35.91	**0.02**	34.72	34.67	35.99	**0.05**	34.77	34.78	34.72	**-0.01**
2014	33.83	33.81	34.21	**0.02**	34.82	34.80	35.23	**0.01**	35.08	34.99	36.52	**0.09**	35.06	35.07	34.94	**-0.01**
2015	33.80	33.80	33.79	**0.01**	35.09	35.08	35.46	**0.01**	35.13	35.06	36.16	**0.07**	35.02	35.05	34.84	**-0.03**
2016	33.90	33.86	34.51	**0.04**	35.08	35.05	37.03	**0.04**	35.17	35.05	36.97	**0.12**	35.12	35.13	35.07	**-0.01**
2017	34.17	34.11	34.97	**0.06**	35.24	35.21	36.46	**0.03**	35.28	35.18	36.71	**0.10**	35.13	35.13	35.19	**0.00**
2018	34.01	33.98	34.48	**0.03**	35.30	35.28	36.12	**0.02**	35.43	35.34	36.70	**0.09**	35.28	35.27	35.19	**0.01**
2019	34.38	34.33	34.99	**0.05**	35.62	35.59	37.00	**0.04**	35.62	35.52	37.04	**0.10**	35.74	35.73	35.19	**0.01**

Notes: PLE (Period Life Expectancy); Total (total resident population); NB (native-born population); FB (foreign-born population)

Source: authors' calculations based upon respective register data for each country

Table 6. Period life expectancy at age 75 women in four Nordic countries, men, 1990-2019.

Year	Denmark, e75				Finland, e75				Norway, e75				Sweden, e75			
	Total	NB	FB	**Total - NB**	Total	NB	FB	**Total - NB**	Total	NB	FB	**Total - NB**	Total	NB	FB	**Total - NB**
1990	10.98	10.99	10.62	**-0.01**	10.53	10.53	10.90	**0.01**	11.10	11.09	11.68	**0.01**	11.42	11.41	11.78	**0.01**
1991	11.14	11.15	10.67	**-0.02**	10.65	10.64	11.12	**0.00**	11.30	11.28	12.34	**0.02**	11.58	11.57	11.72	**0.01**
1992	11.04	11.04	10.97	**0.00**	10.75	10.75	10.34	**-0.01**	11.44	11.43	11.92	**0.01**	11.68	11.66	12.01	**0.02**
1993	10.81	10.82	10.73	**-0.01**	10.47	10.47	10.96	**0.01**	11.18	11.17	11.43	**0.01**	11.52	11.52	11.55	**0.00**
1994	11.05	11.04	11.16	**0.00**	11.00	10.99	11.61	**0.01**	11.64	11.64	11.88	**0.00**	12.02	12.00	12.35	**0.02**
1995	10.87	10.88	10.56	**-0.01**	10.99	10.99	11.23	**0.00**	11.59	11.58	11.98	**0.01**	11.94	11.94	11.98	**0.00**
1996	11.07	11.06	11.46	**0.01**	11.12	11.13	10.92	**0.00**	11.79	11.78	12.26	**0.01**	12.00	11.99	12.25	**0.01**
1997	11.22	11.21	11.21	**0.00**	11.22	11.23	10.83	**-0.01**	11.72	11.70	12.35	**0.02**	12.13	12.14	12.02	**-0.01**
1998	11.46	11.46	11.29	**0.00**	11.43	11.42	12.26	**0.01**	11.82	11.82	12.07	**0.00**	12.20	12.18	12.49	**0.02**
1999	11.24	11.23	11.49	**0.01**	11.46	11.45	12.23	**0.01**	11.77	11.77	11.90	**0.00**	12.07	12.06	12.25	**0.01**
2000	11.46	11.47	11.24	**-0.01**	11.51	11.52	11.47	**0.00**	11.95	11.94	12.40	**0.01**	12.23	12.23	12.34	**0.00**
2001	11.39	11.40	11.18	**-0.01**	11.73	11.74	11.43	**0.00**	12.00	11.99	12.52	**0.01**	12.25	12.26	12.41	**-0.01**
2002	11.29	11.28	11.79	**0.02**	11.69	11.67	13.03	**0.02**	11.95	11.95	11.88	**0.00**	12.18	12.20	12.02	**-0.02**
2003	11.50	11.49	11.71	**0.01**	11.87	11.87	11.58	**0.00**	12.26	12.26	12.33	**0.00**	12.44	12.43	12.66	**0.01**
2004	11.78	11.76	12.23	**0.02**	12.47	12.46	13.14	**0.01**	12.54	12.54	12.55	**0.00**	12.65	12.67	12.61	**-0.02**
2005	11.81	11.81	12.00	**0.01**	12.70	12.69	13.56	**0.01**	12.72	12.71	13.22	**0.01**	12.70	12.72	12.69	**-0.02**
2006	11.81	11.80	12.03	**0.01**	12.80	12.79	13.58	**0.01**	12.61	12.60	12.93	**0.01**	12.76	12.77	12.76	**-0.01**
2007	11.77	11.77	11.82	**0.00**	12.84	12.83	13.32	**0.01**	12.65	12.65	12.86	**0.00**	12.67	12.68	12.67	**-0.01**
2008	12.01	11.99	12.49	**0.02**	12.88	12.88	13.18	**0.00**	12.85	12.84	13.13	**0.01**	12.80	12.83	12.63	**-0.03**
2009	11.95	11.94	12.34	**0.01**	13.04	13.04	13.18	**0.00**	12.89	12.88	13.19	**0.01**	13.03	13.05	12.92	**-0.02**
2010	12.07	12.04	12.72	**0.03**	13.00	12.99	13.47	**0.01**	13.02	13.00	13.71	**0.02**	13.02	13.02	13.15	**0.00**
2011	12.37	12.35	12.93	**0.03**	13.22	13.22	13.50	**0.00**	13.09	13.07	13.83	**0.02**	13.10	13.14	13.01	**-0.04**
2012	12.48	12.47	12.66	**0.01**	13.15	13.14	13.94	**0.01**	13.00	12.97	13.95	**0.03**	12.99	13.00	12.91	**-0.01**
2013	12.55	12.54	12.86	**0.01**	13.29	13.28	13.75	**0.01**	13.21	13.19	14.17	**0.02**	13.14	13.15	13.13	**-0.01**
2014	12.90	12.88	13.34	**0.02**	13.27	13.27	13.16	**0.00**	13.43	13.40	14.27	**0.03**	13.41	13.42	13.38	**-0.01**
2015	12.86	12.88	12.64	**-0.01**	13.37	13.37	13.52	**0.00**	13.39	13.38	13.81	**0.01**	13.32	13.37	13.07	**-0.05**
2016	12.93	12.91	13.35	**0.02**	13.42	13.40	14.85	**0.02**	13.49	13.43	14.77	**0.06**	13.39	13.41	13.35	**-0.02**
2017	12.97	12.94	13.48	**0.03**	13.58	13.57	14.40	**0.01**	13.53	13.51	14.27	**0.02**	13.36	13.36	13.38	**0.00**
2018	12.88	12.87	13.16	**0.01**	13.59	13.58	13.83	**0.00**	13.60	13.58	14.08	**0.02**	13.49	13.50	13.39	**-0.01**
2019	13.16	13.14	13.42	**0.01**	13.85	13.84	14.57	**0.01**	13.72	13.68	14.69	**0.04**	13.81	13.81	13.39	**0.00**

Notes: PLE (Period Life Expectancy); Total (total resident population); NB (native-born population); FB (foreign-born population)

Source: authors' calculations based upon respective register data for each country

Supplementary file 6

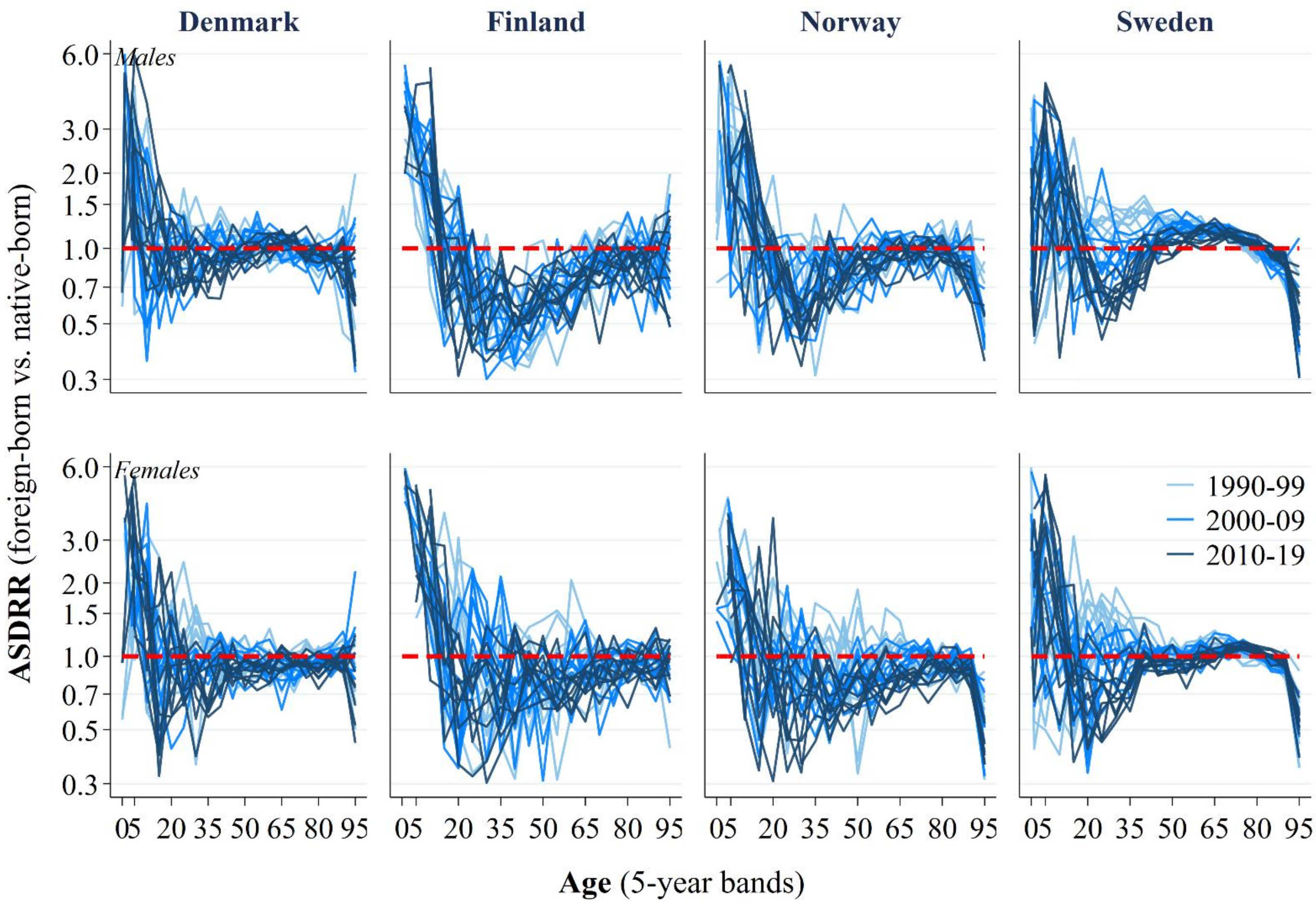

Figure 1. Age-specific death rate ratios, foreign-born relative to native-born, in four Nordic countries, 1990-2019.

Source: authors' calculations based upon respective register data for each country.
